# Cross-feeding between intestinal pathobionts promotes their overgrowth during undernutrition

**DOI:** 10.1038/s41467-021-27191-x

**Published:** 2021-11-25

**Authors:** K. E. Huus, T. T. Hoang, A. Creus-Cuadros, M. Cirstea, S. L. Vogt, K. Knuff-Janzen, P. J. Sansonetti, P. Vonaesch, B. B. Finlay

**Affiliations:** 1 Michael Smith Laboratories and Department of Microbiology and Immunology, University of British Columbia, Vancouver, BC Canada; 2 Unité de Pathogénie Microbienne Moléculaire, Institut Pasteur, Paris, France; 3 Canadian Institute for Advanced Research, Toronto, Ontario Canada; 4 Present Address: Center for Microbes, Development and Health, Institut Pasteur de Shanghai, Shanghai, China; 5 Present Address: Human and Animal Health Unit, Swiss Tropical and Public Health Institute & University of Basel, Basel, Switzerland

**Keywords:** Microbial ecology, Microbiome, Symbiosis, Malnutrition

## Abstract

Child undernutrition is a global health issue associated with a high burden of infectious disease. Undernourished children display an overabundance of intestinal pathogens and pathobionts, and these bacteria induce enteric dysfunction in undernourished mice; however, the cause of their overgrowth remains poorly defined. Here, we show that disease-inducing human isolates of *Enterobacteriaceae* and *Bacteroidales* spp. are capable of multi-species symbiotic cross-feeding, resulting in synergistic growth of a mixed community in vitro. Growth synergy occurs uniquely under malnourished conditions limited in protein and iron: in this context, *Bacteroidales* spp. liberate diet- and mucin-derived sugars and *Enterobacteriaceae* spp. enhance the bioavailability of iron. Analysis of human microbiota datasets reveals that *Bacteroidaceae* and *Enterobacteriaceae* are strongly correlated in undernourished children, but not in adequately nourished children, consistent with a diet-dependent growth synergy in the human gut. Together these data suggest that dietary cross-feeding fuels the overgrowth of pathobionts in undernutrition.

## Abstract (web summary)

Malnourished children experience a high burden of intestinal pathogens that exacerbate growth stunting, and preventing this pathogen overgrowth has proved challenging. Here the authors show that diet-specific bacterial crossfeeding contributes to the overgrowth of intestinal pathogens during child malnutrition.

## Introduction

Malnutrition is a global health issue, responsible for nearly half of all deaths in children under 5 years old^1,2^. In addition to diet, an intestinal disorder known as environmental enteric dysfunction (EED) contributes significantly to child undernutrition in regions with inadequate sanitation. EED is characterized by blunting of the small intestinal villi^3–6^ alongside increased intestinal permeability and inflammation^7–9^. EED is thought to be microbially driven and has been linked to dysbiosis and overgrowth of the microbiota, most notably a high burden of *Proteobacteria* species and asymptomatic carriage of enteropathogens^10–13^. This drives a vicious cycle of nutrient malabsorption and increased susceptibility to infection. However, intervention trials that aimed to provide both an improved diet and improved hygiene in early life failed to reduce the prevalence of bacterial pathogens or to improve stunting or EED^14^. The etiology of EED and the factors driving microbial dysbiosis in these children remain poorly understood.

In 2015, our lab reported that a combination of dietary malnutrition and specific fecal bacteria could replicate the features of EED in mice^15^. Mice fed an isocaloric low-protein, low-fat diet, and iteratively exposed to commensal human *Bacteroidales* and *E. coli* isolates displayed impaired growth, small intestinal villous blunting, and abnormal intestinal permeability and inflammation, analogous to human EED^15^. Another research group independently found that a combination of *Bacteroidetes*, *Enterobacteriaceae*, and *Enterococcus* from children with severe acute malnutrition could induce enteropathy in malnourished gnotobiotic mice^16^. Intriguingly, both models were dependent on the combined presence of both *Bacteroidales* and *Enterobacteriaceae* members rather than any taxon individually, suggesting a pathobiotic synergy between these bacteria. Furthermore, both models were dependent on a carbohydrate-rich malnourished diet: mice that received the same bacterial cocktail but were fed an isocaloric control diet rich in protein and fat did not develop EED^15,16^. The mechanism driving this context-dependent synergy between *Bacteroidales* and *E. coli* is currently unknown. We hypothesized that *Bacteroidales* and *E. coli* were capable of metabolic cross-feeding and that this would be exacerbated in protein-deficient, carbohydrate-rich environments, leading to synergistic growth of these bacteria and intestinal dysbiosis in EED mice.

Metabolic interactions between *Bacteroidales* and *Enterobacteriaceae* strains have been previously shown to contribute to microbial dysbiosis and to the outgrowth of pathogens. *Bacteroides* spp. digest complex polysaccharides in the host diet and host mucosa, liberating simple sugars that act both as growth substrates and as signaling cues for pathogenic *E. coli* and *Salmonella*^17–20^. Conversely, *Bacteroides* spp. have been shown to benefit from the presence of *Enterobacteriaceae* during intestinal and extraintestinal infections, thanks to the production of hemoglobin-degrading proteases and of iron-scavenging siderophores by *Enterobacteriaceae*^21,22^. However, both of these interactions were studied independently, and reciprocal cross-feeding in mixed communities has not been shown. It is further unknown how these interactions are influenced by diet.

Here, we show that EED-inducing strains of *Bacteroidales* spp. and *Escherichia* spp. are capable of mutual, bidirectional cross-feeding, resulting in synergistic growth of a mixed community in vitro. Growth synergy is highly dependent on the nutritional environment and requires the presence of both host mucin and dietary carbohydrates under protein- and iron-limiting conditions. Moreover, the abundances of *Bacteroidaceae* and *Enterobacteriaceae* are strongly correlated in the fecal microbiota of undernourished, but not normally nourished, children. Together these data indicate a role for host nutrition in shaping pathobiont co-dependencies within the microbiota.

## Results

### Synergistic growth of *Bacteroidales* and *E. coli* in co-culture

**Fig. 1:**
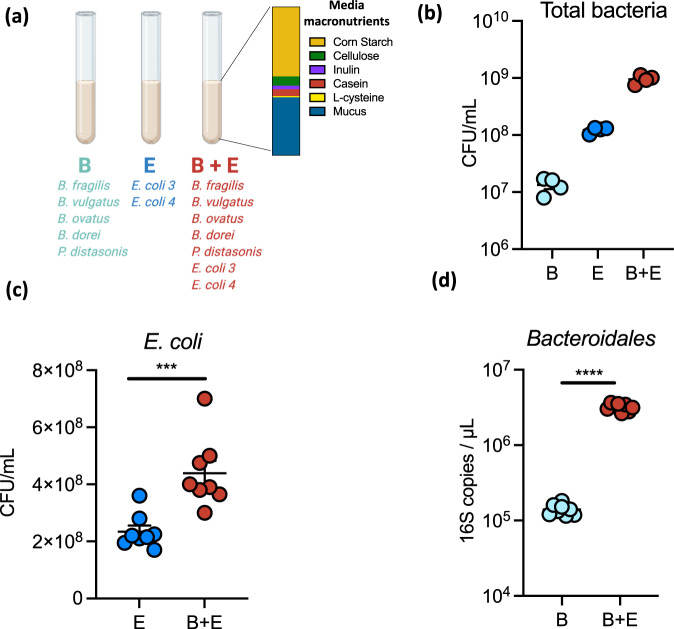
Synergistic growth of *Bacteroidales* and *Enterobacteriaceae* in co-culture. **a** Schematic of the experimental set-up. After a 24 h culture, bacterial endpoint growth was determined for (**b**) the total community (*n* = 4), **c** *E. coli* (*n* = 8), and **d** *Bacteroidales* (*n* = 8). Mean + /− SEM are displayed. Significance was determined by two-sided *t* test. ***P < 0.001; ****P < 0.0001. **c**, **d** B *Bacteroidetes* mix, E *E. coli* mix, CFU colony-forming units. Source data are provided as a Source Data file. Exact *P* values and test statistics are reported in Supplementary Table 8.

We designed a complex medium, designated “MAL-M”, to reflect the macronutrients available in the intestine during chronic protein undernutrition. Dietary plant polysaccharides (starch, inulin, and cellulose) and protein (casein) were included based on their abundance in a malnourished mouse diet previously reported by our group^15^, and mucin was added as a source of host glycoprotein (Fig. 1a and Supplementary Table 1), leading to a medium rich in carbohydrates but deficient in protein and fat. This medium was used to co-culture seven human intestinal isolates used previously to induce inflammation and barrier disruption in our mouse model of malnutrition and EED: five strains of *Bacteroidales* (*Bacteroides fragilis, B. vulgatus, B. ovatus, B. dorei, Parabacteroides distasonis*) and two *Enterobacteriaceae* (*Escherichia coli* 4_1_47 and *Escherichia coli* 3_2_53 (Fig. 1a and Supplementary Table 2). Unless otherwise indicated, in this manuscript *Bacteroidales* will refer to all five strains of *Bacteroidales* grown in combination, while *E. coli* will refer to both strains of *E. coli* grown together (“B” and “E”, respectively, in the figures; Fig. 1a).

Both *Bacteroidales* and *E. coli* were capable of growing in MAL-M, attaining 10^7^ and 10^8^ CFU/mL, respectively, after 24 h of anaerobic growth (Fig. 1b and Supplementary Fig. 1). When all seven species were grown in combination, the co-culture grew to 10^9^ total CFU/mL, a tenfold increase that was substantially more than expected based on simple additive growth (Fig. 1b).

The synergistic increase in bacterial CFU was driven by improved growth of both *Bacteroidales* and *E. coli* simultaneously. The growth of *E. coli* roughly doubled in the presence of *Bacteroidales* (Fig. 1c), while the growth of *Bacteroidales* increased by tenfold or more in the presence of *E. coli* (Fig. 1d). These bacteria thus mutually support one another’s growth through cooperative interactions.

### Synergistic growth is dependent on the nutritional environment

To better understand the importance of the nutritional environment in promoting *Bacteroidales–E. coli* growth synergy, we compared growth in the original MAL-M medium with medium in which the weight-based ratio of protein to carbohydrate was reversed (“CON-M”). Bacteria were also grown in CON and MAL media in the absence of host mucin (Supplementary Fig. 2A and Supplementary Table 1). In these complex media, the presence of mucin promoted the total growth of the co-culture (Supplementary Fig. 2B). However, only the carbohydrate-rich mucin medium (MAL-M) led to enhanced growth of both *Bacteroidales* and *E. coli* simultaneously compared to their respective monocultures (Supplementary Fig. 2C, D).

**Fig. 2:**
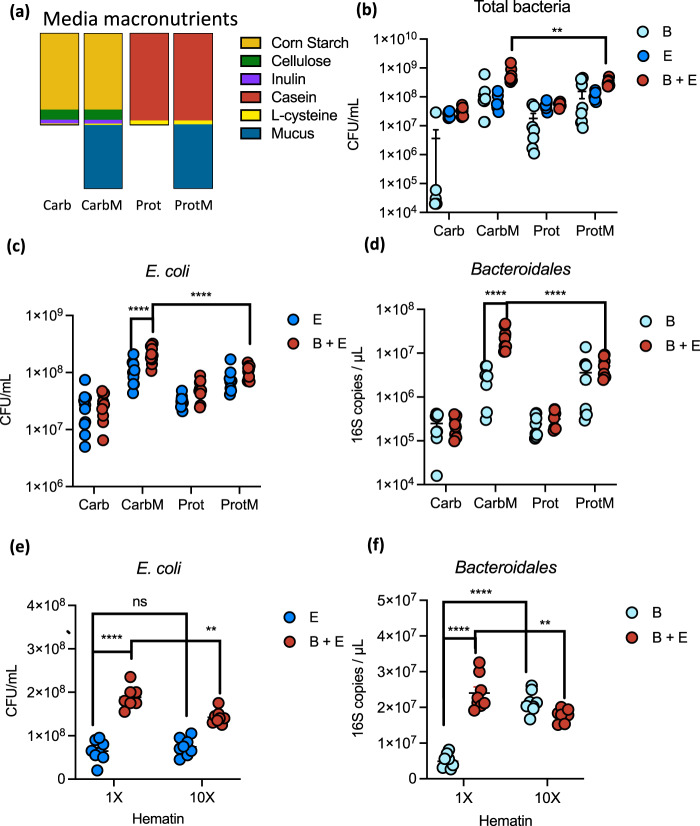
Synergistic growth of *Bacteroidales* and *Enterobacteriaceae* is dependent on the nutritional environment. **a** Media macronutrient compositions. After a 24 h culture, bacterial endpoint growth was determined for (**b**) the total community, **c** *E. coli* and **d** *Bacteroidetes* (*n* = 8). **e**, **f** Growth of (**e**) *E. coli* and (**f**) *Bacteroidales* in MAL-M media with 1× (1.9 µM) or 10× (19 µM) hematin (*n* = 8). Mean + /− SEM are displayed. Significance was determined by two-way ANOVA with Tukey’s post hoc test (**b**–**f**). **P value < 0.01, ****P value < 0.0001. B *Bacteroidales* mix, E *E. coli* mix, CFU colony-forming units, Carb carbohydrate media, CarbM carbohydrate plus mucus media, Prot protein media, ProtM protein plus mucus media. Source data are provided as a Source Data file. Exact *P* values and test statistics are reported in Supplementary Table 8.

Substitution of casein with whey as the protein source in MAL-M media also resulted in similar reciprocal growth advantages for both *Bacteroidales* and *E. coli* (Supplementary Fig. 2E, F). To more clearly distinguish the importance of carbohydrate versus protein in promoting synergistic growth, we cultured bacteria in media containing only dietary carbohydrates (inulin, starch, and cellulose; “Carb”) or only dietary protein (casein; “Prot”) as the carbon source, with or without host mucin (Fig. 2a and Supplementary Table 1; “CarbM” and “ProtM” designate mucin-containing media). Importantly, when grown separately, *Bacteroidales* and *E. coli* each showed similar growth in the CarbM compared to the ProtM media, indicating that the inherent availability of nutrients is comparable between these two media (Fig. 2b–d). However, total bacterial counts in co-culture were significantly higher in the carbohydrate- and mucus-rich CarbM medium than in any other condition, with both *E. coli* and *Bacteroidales* showing a distinct advantage in CarbM co-culture compared to ProtM co-culture (Fig. 2b–d).

Iron is a key micronutrient that is frequently limited in undernourished diets, and *Bacteroidales* are known to be particularly dependent on organic iron sources^23^. To investigate the impact of iron bioavailability, we cultured bacteria in the original MAL-M medium (containing 1.9 µM hematin) or in a medium where organic iron was provided at tenfold excess (19 µM hematin). *Bacteroidales*, but not *E. coli*, grew substantially better in the hematin-enriched medium (Fig. 2e, f). Strikingly, however, excess hematin significantly dampened the synergistic advantage of both *Bacteroidales* and *E. coli* when grown in co-culture (Fig. 2e, f). Although the hematin was dissolved in a histidine solution to improve its stability and bioavailability, a control condition supplemented with histidine only did not impact *Bacteroidales* growth (Supplementary Fig. 2G). These data suggest that organic iron is a limiting nutrient for *Bacteroidales* growth in MAL-M medium, and that iron bioavailability affects synergistic growth in co-culture.

Together, these data support the importance of the nutritional environment in *Bacteroidales–E. coli* synergism. *Bacteroidales* and *E. coli* experience a mutual growth advantage under protein- and iron-limited conditions in the presence of mucus and dietary carbohydrates.

### Competitive and cooperative interactions contribute to strain abundance in the mixed community

**Fig. 3:**
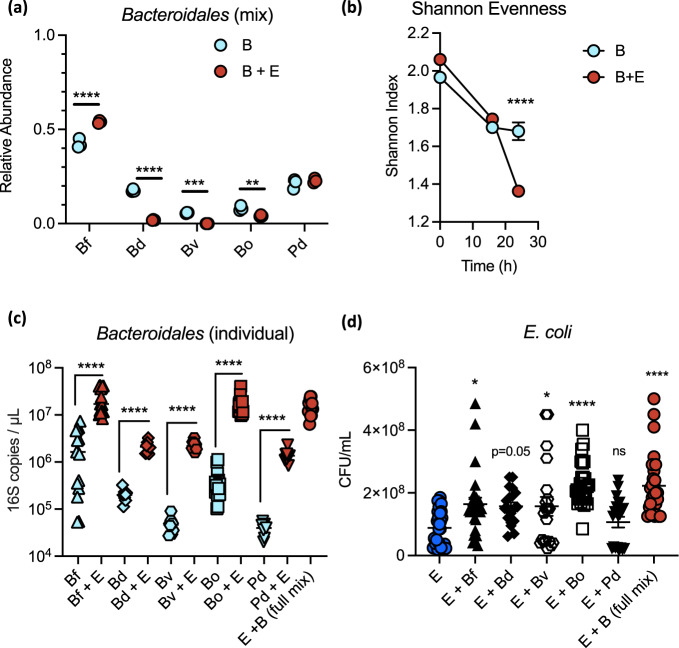
Competitive and cooperative interactions contribute to strain abundance in the mixed community. **a** Relative abundance of each *Bacteroidales* species at 24 h endpoint, in a mixed community with or without *E. coli* (*n* = 3). **b** Shannon evenness of the communities over time (*n* = 3). **c** Growth of each individual *Bacteroidales* at 24 h endpoint, alone or in co-culture with *E. coli* (*n* = 8 for Bd, Bv, Pd; *n* = 15 for Bf, Bo, E + B). **d** Growth of *E. coli* at 24 h endpoint with or without each individual *Bacteroidales* species or the full mix of all five *Bacteroidales* (*n* = 20 for Bd, Bv, Bd; *n* = 27 for Bf, Bo, E + B). Mean + /− SEM. Significance was determined by two-way ANOVA with Sidak’s post hoc test (**a**, **b**) or Dunnett’s post hoc test (**d**). In (**c**) each strain comparison was independent of the others; significance was determined by repeated two-sided *t* test with FDR correction. *P value < 0.05, **P value < 0.01, ***P value < 0.001, ****P value < 0.0001. B full *Bacteroidales* mix, E *E. coli* mix, Bf *B. fragilis,* Bo *B. ovatus,* Bv *B. vulgatus,* Pd *P. distasonis*, Bd *B. dorei*. Source data are provided as a Source Data file. Exact *P* values and test statistics are reported in Supplementary Table 8.

To determine whether the expansion of *Bacteroidales* was driven by the abundance of one or more of the individual species present in our cocktail, we performed 16S rRNA sequencing of *Bacteroidales* cultures in MAL-M media in the presence or absence of *E. coli*. Relative abundances were converted to total abundances by means of *Bacteroidetes-*specific 16S rRNA qPCR. All five *Bacteroidales* strains were able to grow in co-culture in the absence of *E. coli* (Supplementary Fig. 3A–E). However, *B. fragilis* was by far the dominant taxon by relative abundance; moreover, in the presence of *E. coli*, *B. fragilis* bloomed at the expense of the other *Bacteroides* (Fig. 3a and Supplementary Fig. 3A–E). Indeed, after 24 h of growth in co-culture, *B. fragilis* constituted over 50% of the *Bacteroidales* by relative abundance and *B. vulgatus* was undetectable. Community richness and evenness, as represented by the Shannon index, were thus significantly decreased in co-culture by 24 h, despite the greater initial diversity of this mixture (Fig. 3b). Notably, although *B. ovatus* relative abundance was decreased in the presence of *E. coli* by 24 h (Fig. 3a), *B. ovatus* showed only a mild defect in total abundance at 24 h (Supplementary Fig. 3C), and in fact showed improved total growth in the presence of *E. coli* at the earlier 16 h timepoint, suggesting it might simply experience an earlier benefit than the other strains.

To further delineate strain interactions, we tested growth pairs of *E. coli* with each individual *Bacterodales* spp. In contrast to the results from a mixed culture, all individual *Bacteroidales* benefited substantially from pairwise co-culture with *E. coli* (Fig. 3c). This suggests that all strains tested here are capable of cross-feeding with *E. coli*, but that competition with other *Bacteroidales* restricts this benefit in more complex communities. Indeed, when growth pairwise with *E. coli*, both *B. fragilis* and *B. ovatus* were capable of reaching similar total biomass to the entire *Bacteroidales* co-culture (Fig. 3c).

Reciprocally, all *Bacteroides* strains appeared to confer a modest growth advantage to *E. coli* in pairwise co-culture, but to varying degrees (Fig. 3d). There was notable batch variation in the magnitude of bacterial growth, possibly due to media batch effects or minor timing differences; however, trends were consistent between replicates (Supplementary Fig. 3F–H). *B. ovatus* provided the most consistent and significant individual growth benefit to *E. coli*, largely recapitulating the effect of the complete community (Fig. 3d and Supplementary Fig. 3F–H). Nevertheless, removing *B. ovatus* from the complete mix still resulted in a substantial co-culture benefit for *E. coli* (Supplementary Fig. 3H), suggesting that the contributions of the other taxa are sufficient in combination to provide a growth advantage to *E. coli*.

Together these data indicate that many different members of the *Bacteroidales* order are capable of cooperation with *E. coli* during nutrient-limited conditions. In the presence of other *Bacteroidales*, both cooperation and competition contribute to the emergent growth of the community.

### *Bacteroidales* and *E. coli* exchange soluble metabolites

**Fig. 4:**
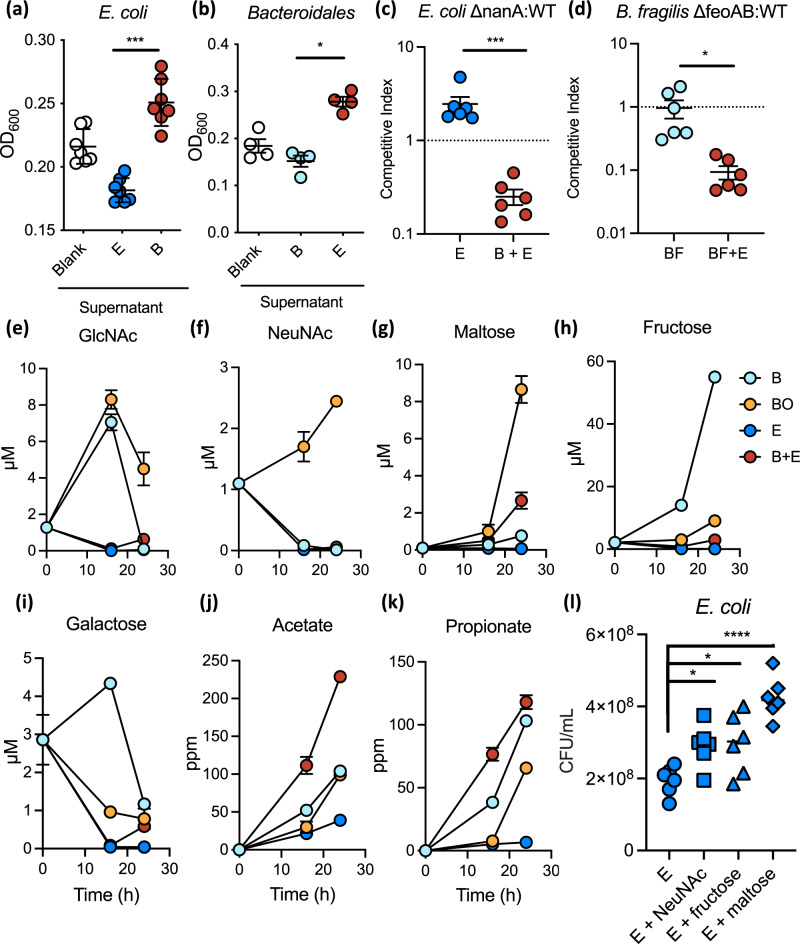
*E. coli* and *Bacteroidales* exchange soluble metabolites, including sialic acid and heme. **a**, **b** Growth of *E. coli* (*n* = 7) (**a**) and *Bacteroidales* (*n* = 4) (**b**) at 24 endpoint, as determined by optical density at 600 nm (O.D.600) in the sterile-filtered supernatants of blank media (“Blank”), a 24 h *E. coli* culture (“E”), or a 24 h *Bacteroidales* culture (“B”). **c** Competitive index of ∆*nanA E. coli*, deficient in sialic acid metabolism, compared to wild-type in the presence or absence of *Bacteroidales* (*n* = 6). **d** Competitive index of ∆*feoAB B. fragilis*, deficient in iron uptake, compared to wild-type strain 638 R in the presence or absence of *E. coli* (*n* = 6). **e**–**k** Metabolite abundance in sterile-filtered bacterial culture supernatants over time (*n* = 3). Mean + /− SEM are displayed: **e** N-acetylglucosamine (GlcNAc); **f** N-acetylneuraminic acid (NeuNAc, sialic acid); **g** maltose; **h** fructose; **i** galactose; **j** acetate; **k** propionate. **l** Growth of *E. coli* in MAL-M media supplemented with 1 mM of free sugars (*n* = 6). Significance was determined by ANOVA with post hoc Dunnett’s test (**a**, **b**, **l**), or by two-sided *t* test (**c**, **d**). *P value < 0.05, **P value < 0.01, ***P value < 0.001. B *Bacteroidales* mix, BF *B. fragilis*, BO *B. ovatus,* E *E. coli* mix. Source data are provided as a Source Data file. Exact *P* values and test statistics are reported in Supplementary Table 8.

To determine whether growth synergism in co-culture was mediated by soluble factors, we assessed bacterial growth in sterile supernatants from blank media or from 24-h cultures of *Bacteroidales* or *E. coli* in MAL-M. As expected, *E. coli* grew significantly better in supernatants from *Bacteroidales* culture compared to blank media or its own spent growth media, while *Bacteroidales* grew significantly better in *E. coli* culture supernatants compared to blank media or its own spent growth media (Fig. 4a, b and Supplementary Fig. 4A, B). Thus, both *E. coli* and *Bacteroidales* produce soluble factors in carbohydrate-rich environments that benefit the other community member.

To identify metabolic pathways involved in synergistic growth, we began by testing the role of mucin-derived sugars, since mucus stimulated co-culture growth (Fig. 2b and Supplementary Fig. 2B) and has been previously shown to fuel microbial cross-feeding^17,18^. Indeed, co-culture of *E. coli* with *B. thetaiotaomicron*, a species previously shown to cooperatively liberate sialic acid from mucus^17^, gave a dramatic growth benefit to these *E. coli* strains (Supplementary Fig. 4C). We, therefore, generated knockout strains of *E. coli* lacking key metabolic enzymes for consumption of sialic acid (∆*nanA*) and fucose (∆*fucI*), two sugars that decorate mucin (Supplementary Table 2). These mutant strains were completely unable to grow on sialic acid or fucose, respectively (Supplementary Fig. 4D, E) but showed a growth advantage compared to wild-type *E. coli* on glucose (Supplementary Fig. 4F), possibly because there is a metabolic cost to producing these enzymes in the absence of a substrate.

*E. coli* ∆*nanA* strains were at a significant competitive disadvantage compared to wild-type *E. coli* in MAL-M media during *Bacteroidales* co-culture, but were not at a disadvantage when grown alone (Fig. 4c). Further supporting the importance of carbohydrate-rich nutritional context that we identified earlier, when ∆*nanA E. coli* was co-cultured in the Carb and Prot media described above, we only observed a significant competitive defect of ∆*nanA E. coli* in CarbM media (Supplementary Fig. 4G). There was no significant competitive defect for Δ*nanA* in ProtM media, even though this condition contained identical quantities of mucin as a source of sialic acid, which is consistent with the lack of co-culture growth advantage in this media (Fig. 2b–d). We did not observe any competitive defect for the ∆*fucI* mutant in co-culture (Supplementary Fig. 4H). Together these data suggest that sialic acid metabolism contributes to *E. coli* growth in the presence of *Bacteroidales*, and that this interaction is specifically enhanced in a polysaccharide- and mucin-rich environment.

*B. fragilis* has been shown to benefit from *E. coli* during extraintestinal co-infection due to *E. coli* liberation of heme from hemoglobin^21^. *E. coli* also produces siderophores which can be co-opted by *Bacteroides* for iron acquisition^22^ and *E. coli* supports porphyrin-auxotrophic *Bacteroides* in co-culture^24^. We confirmed that the *E. coli* strains used in this study also produce siderophores under iron-limiting conditions (Supplementary Fig. 5A). We further investigated the importance of iron using *B. fragilis* as a proof-of-principle strain. Similar to total *Bacteroidales* growth, the growth of *B. fragilis* was significantly enhanced by hematin supplementation (Supplementary Fig. 5B). Growth was not dependent on the concentration of vitamin B12, another putatively limiting micronutrient (Supplementary Fig. 5C). To further test whether iron was responsible for the *B. fragilis* growth advantage in co-culture, we used a *B. fragilis* ∆*feoAB* knockout strain deficient in both ferrous iron and heme uptake^25^ and compared it to parent strain *B. fragilis* 638R (Supplementary Table 2). Similar to the *B. fragilis* strain 3/1/12 used elsewhere in this paper (and indeed to all *Bacteroides* spp. tested here), *B. fragilis* 638R experienced a significant growth advantage in MAL-M media in the presence of *E. coli* (Supplementary Fig. 5D). Further, ∆*feoAB B. fragilis* was at a competitive disadvantage compared to its parent strain, but only when co-cultured in the presence of *E. coli* (Fig. 4d). These data suggest that *B. fragilis* benefits from *E. coli*-derived iron.

Both ∆*nanA E. coli* and ∆*feoAB B. fragilis* still benefited from co-culture when grown without a wild-type competitor, suggesting that other unidentified factors also contribute to growth synergy (Supplementary Fig. 5D, E). To identify other potential nutrient sources in co-culture, we quantified a targeted panel of saccharides and short-chain fatty acids in bacterial supernatants using mass spectrometry. In addition to the complex mixes of *Bacteroidales, E. coli*, and co-cultured bacteria, we included *B. ovatus* as a proof-of-principle strain due to its ability to provide a substantial individual benefit to *E. coli* (Fig. 3d). These results indicate that a large number of mucin- and carbohydrate-derived sugars are generated by the full *Bacteroidales* mix, including N-acetylglucosamine, fructose, galactose, xylose, and fucose (Fig. 4e–i and Supplementary Fig. 6A–L). *B. ovatus* was independently capable of generating many of these same sugars and was also a net producer of sialic acid and maltose (Fig. 4e–g). Importantly, all of these sugars were depleted in *E. coli* monoculture relative to baseline, suggesting that *E. coli* consumes them; further, they were largely depleted in *Bacteroidales–E. coli* co-culture (Fig. 4e–i and Supplementary Fig. 6A–L). In contrast, the short-chain fatty acids acetate and propionate, two end products of microbial fermentation, accumulated in *Bacteroidales–E. coli* co-culture (Fig. 4j, k). The growth of *E. coli* was enhanced in MAL-M media supplemented with free sialic acid, fructose, or maltose, supporting the ability of any or all of these sugars to provide a growth advantage to *E. coli* during cross-feeding (Fig. 4l).

Together these data identify a large number of possibilities for carbohydrate-based cross-feeding between *Bacteroidales* and *E. coli*, and suggest efficient utilization of shared resources in the mixed community.

### *Bacteroidaceae* and *Enterobacteriaceae* are co-abundant in the fecal microbiota of undernourished children

We hypothesized that if the interbacterial synergism between *Bacteroidales* and *Enterobacteriaceae* occurs in the human intestine, members of these taxa should be co-abundant in the microbiota of undernourished children, whose diets are typically rich in carbohydrates but poor in sources of animal protein. To test this prediction, we analyzed 16S rRNA sequencing data from fecal samples of children in low- and middle-income countries. Data were retrieved from four studies spanning five countries (Central African Republic, India, Madagascar, Malawi, and Peru) and included publicly available data as well as new sequences generated through the Afribiota study^11,12,26–28^. Children were included for analysis if they were 2–5 years of age and had linear growth measurements available as an outcome. Linear growth stunting, a reflection of chronic undernutrition, was defined by height-for-age z-score as detailed in “Methods”.

**Fig. 5:**
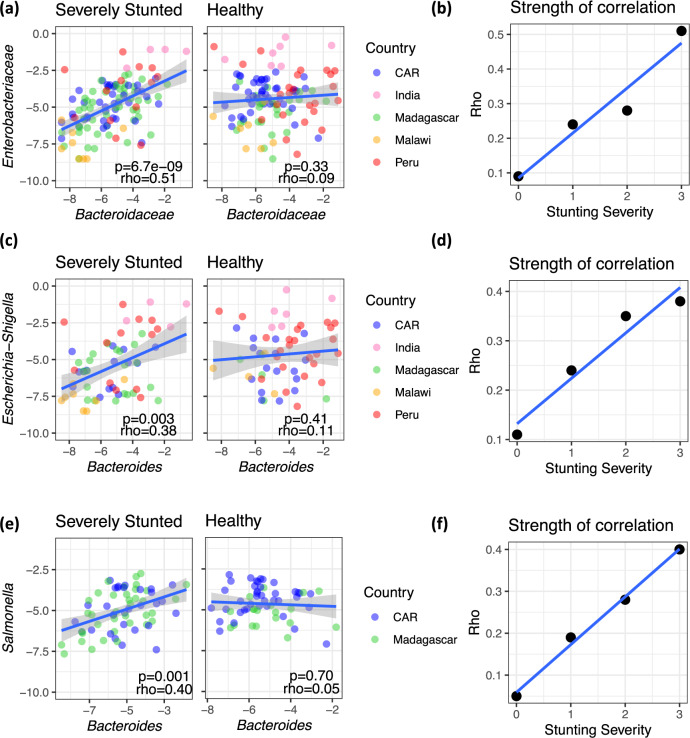
*Bacteroidaceae* and *Enterobacteriaceae* are co-abundant in the fecal microbiota of undernourished children. **a** Correlation between the log-transformed relative abundance of *Bacteroidaceae* and *Enterobacteriaceae* in severely stunted (*n* = 115) and healthy children (*n* = 110). **b** The coefficient of correlation (Spearman’s rho) as calculated between *Bacteroidaceae* and *Enterobacteriaceae* for each subset of stunting severity. **c** Correlation between the log-transformed relative abundance of *Bacteroides* and *Escherichia–Shigella* in severely stunted (*n* = 61) and healthy children (*n* = 56). **d** The coefficient of correlation (Spearman’s rho) as calculated between *Bacteroides* and *Escherichia–Shigella* for each subset of stunting severity. **e** Correlation between the log-adjusted relative abundance of *Bacteroides* and *Salmonella* in severely stunted (*n* = 67) and healthy children (*n* = 59). **f** The coefficient of correlation (Spearman’s rho) as calculated between *Bacteroides* and *Salmonella* for each subset of stunting severity. All panels report two-sided Spearman’s rho and *P* value and exclude children for which one or both taxa were absent. The blue lines in (**a**–**f**) are a linear fit and the shaded areas in (**a**, **c**, and **e**) represent 95% confidence interval. Stunting is defined as follows: 3, “severely stunted” (HAZ ≤ −3); 2, “moderately stunted” (−3>HAZ ≤ −2); 1, “at risk of stunting” (−2>HAZ ≤ −1), and 0, “healthy” (HAZ > −1).

Consistent with our prediction, the relative abundance of the *Bacteroidaceae* and *Enterobacteriaceae* families were significantly positively correlated in the microbiota of stunted children, but not of nonstunted children (Table 1 and Fig. 5a). Moreover, the association between these taxa became progressively stronger with degree of stunting severity (Fig. 5b), supporting the importance of nutritional status in modulating this association. The correlation between *Bacteroidaceae* and *Enterobacteriaceae* in stunted children was consistent in four out of the five countries individually, as well as in the pooled analysis (Table 1); the only exception was the dataset from Dinh et al.^28^, where the sample size after exclusion was very small (*n* = 8). The distribution of stunting was not different between the studies (*P* > 0.1 by ANOVA), and the pooled analysis in stunted children remained significant in a linear model correcting for study effect (*P* = 0.037). Age was also not different between stunted and nonstunted children (*P* > 0.1 by *t* test) and the taxa correlation remained significant in a linear model correcting for both study effect and age (*P* = 0.039).

**Table 1:** Summary of two-sided Spearman’s correlations between *Bacteroidaceae* and *Enterobacteriaceae* log-adjusted relative abundance in 16S rRNA sequencing datasets, in samples where both taxa are present.

Dataset	Country	Nutritional status	N	Rho	*P*
Desai et al.^27^	Malawi	All	41	0.29	0.0636
		Stunted	15	0.50	0.0580
		Nonstunted	26	0.21	0.3126
Dinh et al.^28^	India	All	19	0.62	**0.0051**
		Stunted	8	−0.40	0.3268
		Nonstunted	9	0.52	0.1618
Rouhani et al.^11^	Peru	All	168	0.06	0.4582
		Stunted	69	0.25	**0.0415**
		Nonstunted	99	−0.05	0.6384
Vonaesch et al.^12,26^	Madagascar	All	180	0.26	**0.0005**
		Stunted	92	0.30	**0.0042**
		Nonstunted	88	0.19	0.0780
Vonaesch et al.^12,26^	CAR	All	134	0.20	**0.0184**
		Stunted	60	0.30	**0.0191**
		Nonstunted	74	0.10	0.3606
Meta-analysis	All	All	547	0.29	**4.6e-12**
		Stunted	243	0.39	**1.6e-10**
		Nonstunted	299	0.19	**0.0009**

Bold values indicate statistical significance *p* < 0.05.

Children were defined as either stunted (height-for-age *z*-score ≤ −2) or nonstunted (height-for-age *z*-score > −2) for the analyses reported in this table.

In Madagascar and the Central African Republic, upper intestinal (duodenal and gastric) samples from stunted children were additionally sequenced; the taxonomic correlations in this intestinal site were much less striking (duodenal: *n* = 140, *P* = 0.0958, rho=0.14; gastric: *n* = 61, *P* = 0.3493, rho=0.12). *Bacteroidaceae* spp. are primarily colonic bacteria; thus, the specificity of this relationship to fecal samples likely reflects growth in their preferred niche.

At the genus level, *Bacteroides* were strongly correlated with *Escherichia–Shigella* in feces in a nutritional status-dependent manner (Fig. 5c, d). *Bacteroides* were also strongly correlated with *Salmonella* in stunted children from the Afribiota dataset (Fig. 5e–h). Like *E. coli*, *Salmonella* has been previously shown to benefit from the presence of *B. thetaiotaomicron*. We confirmed that, like our *E. coli* strains, *S. enterica* Typhimurium benefited from the presence of the full *Bacteroidales* mix (Supplementary Fig. 7A) and provided a reciprocal advantage to *Bacteroidales* (Supplementary Fig. 7B) in MAL-M media. *Salmonella* was not detected in the other human studies that we analyzed, and other *Enterobacteriaecae* that were sporadically detected across studies (*Klebsiella, Citrobacter, Enterobacter*, and *Providencia*) were not significantly associated with *Bacteroides* in any of the datasets.

Linear growth stunting can be the result of many different dietary insufficiencies. To explicitly test the importance of iron insufficiency, we compared taxonomic correlations by stunting and anemia in children from Madagascar and CAR (the only populations for which anemia data was available). In this sub-population of two countries, the taxonomic correlation was again modulated by stunting status (stunted children: *n* = 152, *P* = 0.0002, rho = 0.29; nonstunted children: *n* = 162, *P* = 0.2175, rho = 0.10). Interestingly, however, *Bacteroiaceae* and *Enterobacteriaceae* were also significantly correlated in nonstunted anemic children (*n* = 44, *P* = 0.03, rho=0.32). When malnourished children were defined as either stunted or anemic, the association between these taxa was highly significant (*n* = 196, *P* = 9.5e-06, rho = 0.31), while the slight trend seen in nonstunted children disappeared entirely in children who were neither stunted nor anemic (*n* = 112, *P* = 0.9925, rho = −0.001). The modulation of this relationship by anemia is consistent with our in vitro observations that iron limitation contributes to co-dependencies between *Bacteroidaceae* and *Enterobacteriaceae*.

To further investigate the relationship between *Bacteroidaceae* and *Enterobacteriaceae* members at a species and functional level, we gathered publicly available fecal metagenomics data for stunted and nonstunted children 2–5 years old. We identified and processed datasets meeting these criteria from China^29^ and Zimbabwe^30^. Sample sizes after exclusion of younger children and of children without either *Bacteroidaceae* or *Enterobacteriaceae* present in the sample were modest (*N* = 7 and 13 children, respectively, from China and Zimbabwe). Encouragingly, however, correlations between *Bacteroidaceae* and *Enterobacteriaceae* abundance in this small metagenomics dataset were consistent with those observed previously (Supplementary Table 3) and reached significance in stunted children from Zimbabwe and in the combined metagenomics abundance data (Supplementary Table 3; *N* = 20, rho = 0.47, *P* = 0.036). We, therefore, proceeded with an exploratory analysis of species- and gene-level correlations in the pooled metagenomics dataset.

At the species level, the *Bacteroides* most strongly correlated with *E. coli* abundance were *B. ovatus* (rho = 0.48, *P* = 0.031) and *B. dorei* (rho = 0.44, *P* = 0.049). Indeed, as expected based on the family- and genus-level correlations, the majority of *Bacteroides* species were positively correlated with *E. coli*, and many of these species-level associations were consistent between datasets (Supplementary Table 4). These correlations should be interpreted with caution due to the small sample sizes; however, they suggest that in complex human microbiotas, *B. ovatus* and *B. dorei* cooperate particularly successfully with *E. coli*. This is consistent with our in vitro experiments in which both of these species showed cooperative individual growth with *E. coli*.

To explore gene-level associations, we binned all genes from the positively correlated *Bacteroides* species in Supplementary Table 4, reasoning there may be functional redundancy within this taxonomic group, and searched for any *Bacteroides* gene that correlated significantly with the abundance of *E. coli* or *Enterobacteriaceae*. Interestingly, an iron storage protein from the *Bacteroides* group was amongst the five genes correlated (raw *P* value <0.05) with *Enterobacteriaceae* abundance (Supplementary Table 5). A larger number of genes were correlated with *Escherichia* abundance at the genus level, including multiple glycosidases for starch, cellulose, and host N-glycan degradation, and several enzymes involved in B vitamin biosynthesis (Supplementary Table 6). Reciprocally, the only *E. coli* gene correlated with *Bacteroides* or *Bacteroidaceae* abundance was succinyl-diaminopimelate desuccinylase, a gene involved in both amino acid and cell wall biosynthesis (Supplementary Table 5). Although these correlations should all be interpreted with caution given the small sample size and a large number of genes (which prohibited correction for multiple testing), the functions identified are consistent with an exchange of nutrients between these taxa in malnourished environments.

**Fig. 6:**
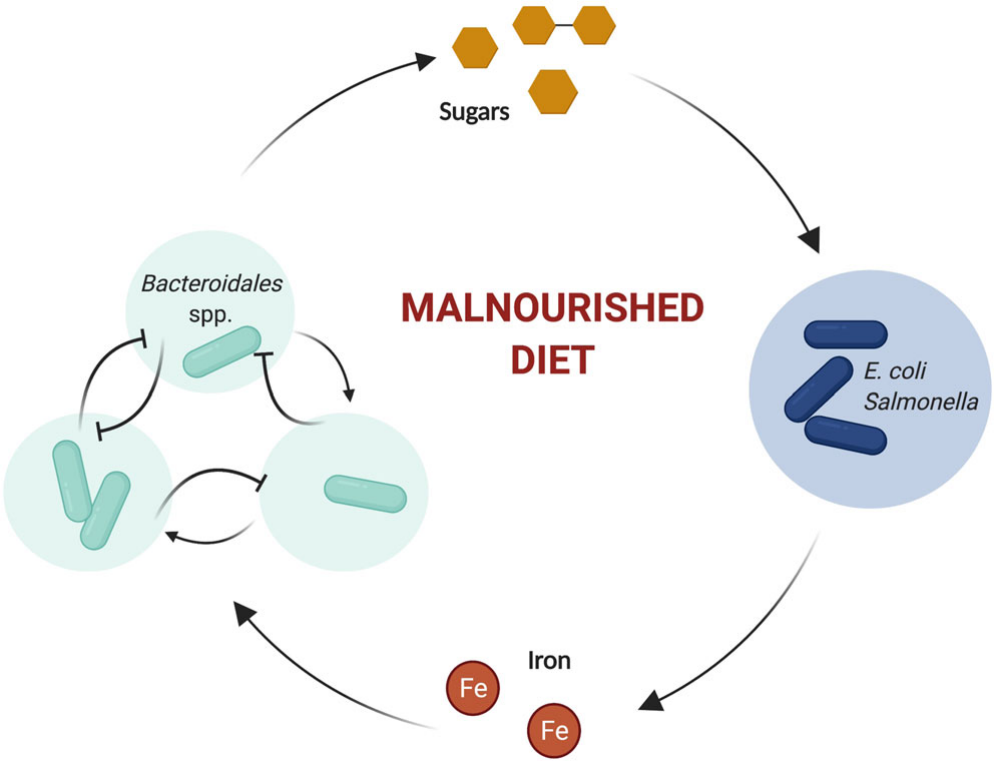
Schematic of the metabolic network between *Bacteroidales* and *Enterobacteriaceae*. In the context of a carbohydrate-rich, protein-deficient malnourished diet, *Bacteroidales* spp. cooperatively liberate sugars. *Enterobacteriaceae* members consume these sugars and provide iron to *Bacteroidales*. Together these interactions constitute a feedback loop leading to enhanced growth of both *Enterobacteriaceae* and *Bacteroidales*. These taxonomic groups contain notable pathogens, including *S. enterica*, enteroaggregative *E. coli*, and enterotoxin-producing *B. fragilis*, which may adversely affect intestinal health.

Taken together, our data suggest that *Bacteroidaceae* and *Enterobacteriaceae* co-vary in the fecal microbiota of undernourished but not normally nourished children, consistent with the ability of these taxa to grow synergistically in carbohydrate-rich, protein-deficient environments (Fig. 6). Importantly, this relationship extends to pathogenic *Enterobacteriaceae* such as *Salmonella*. Moreover, it is highly consistent across independent human populations from seven different countries spanning three continents. This association may therefore be relevant to the growth of pathogens and pathobionts in the undernourished human intestine.

## Discussion

Microbial communities are built from complex ecological interactions. Many of these interactions are negative: bacteria compete with one another and take advantage of their neighbors in the battle for scarce resources^31,32^. This high level of competition within the gastrointestinal microbiota is beneficial to the host, by maintaining relatively stable and diverse communities and by competitively excluding pathogens^31–33^. In contrast, bacterial cooperation is a relatively unusual ecological interaction with predicted destabilizing effects on microbial communities. By mutually enhancing one another’s growth, cooperative bacteria are expected to ‘boom and bust’ together, leading to unstable and low-diversity communities^31^ which are susceptible to external species invasion^34^. Such low-diversity communities are generally considered to be the hallmark of dysbiosis in gut microbiota research, particularly when the high-abundance taxa are facultatively anaerobic *Proteobacteria*^35^. Here we find that a mixture of *Bacteroidales* and *Enterobacteriaceae* are capable of mutually cooperative, synergistic growth during undernutrition, leading to the outgrowth of a low-diversity community (Fig. 6).

The nutritional context was key to the emergence of synergistic growth in these bacteria: co-culture synergy required the presence of both host mucin and dietary carbohydrates, which *Bacteroidales* utilized to liberate a wide range of simple sugars. In contrast, synergistic growth was limited when protein or organic iron was abundant. Growth conditions favoring co-dependency are thus more likely to occur in the intestinal microbiota during protein undernutrition, where there is a paucity of dietary protein and iron compared to plant-based carbohydrates^1,15^. Indeed, we found that *Bacteroidaceae* and *Enterobacteriaceae* were strongly associated in the fecal microbiota of stunted or anemic children, but not in normally nourished children, indicating a nutrient-dependent co-abundance pattern in the human intestine.

Increased abundance of *Enterobacteriaceae* members has been associated with child undernutrition across multiple studies^10–13,36,37^. Importantly, overgrowth of both *Enterobacteriaceae* and *Bacteroidales* has significant potential to contribute to intestinal damage and growth stunting. The murine experiment for this has already been done, showing that intestinal inflammation and exacerbated growth stunting occur in malnourished mice exposed to the same community of *Bacteroidales* and *E. coli* used in this study^15^. Healthy control mice exposed to these bacteria, or malnourished mice exposed only to *Bacteroidales* or to *E. coli* alone, did not develop intestinal damage. Members of the *Bacteroidales* have been repeatedly associated with a lean, non-obese phenotype in Western individuals^38–40^ and alleviate obesity in mice fed high-fat diets, by decreasing total energy harvest and by influencing host appetite and metabolism^41^. These same characteristics could be harmful to undernourished individuals struggling to gain weight. Moreover, in stunted children, *B. fragilis*, *E. coli, Salmonella*, and other members of the *Enterobacteriaceae* frequently carry virulence factors that contribute to intestinal inflammation and damage, thereby exacerbating growth stunting^10,16,36,42^.

It was recently shown that metabolic cross-feeding between intestinal commensals in *Drosophila* supported a mutual growth advantage for bacteria on malnourished diets, with the benefit for host reproduction^43^. The authors suggested a “buffering” effect whereby cross-feeding between commensals allows them to remain resilient to dietary insufficiencies^43^. As noted above, however, cooperative microbial communities also tend to be low-diversity and more susceptible to the invasion of novel species. Indeed, deliberate caloric restriction in humans was recently shown to reduce colonization resistance, leading to the expansion of *C. difficile*^44^. The fecal microbiota of malnourished children been found to include an overabundance of oral and upper airway bacteria, as well as a chronic overabundance of pathogens^12^; these patterns are consistent with reduced colonization resistance of the microbial ecosystem during child malnutrition, and we suggest this might be driven in part by destabilizing cooperative growth. Notably, an intervention trial that administered resistant-starch prebiotics to children in Malawi found that the intervention caused an increase in the fecal inflammatory marker calprotectin, as well as intestinal blooms in fecal *Bacteroidetes*, LPS biosynthesis, and specific *E. coli*-associated enzymes^45^. Although these results were unexpected and unfortunate, they are consistent with our prediction that an overabundance of fermentable fiber in malnourished children can exacerbate *Bacteroides*-*E. coli* cooperative growth and lead to intestinal inflammation.

It has been previously shown that certain *Bacteroides* spp. can support the growth of pathogenic *E. coli* and *Salmonella*^17,18^, and that certain *E. coli* strains can support *Bacteroides* growth^21,22^; however, the possibility for bidirectional feedback has never been explored. We find that many different *Bacteroidales* members are capable of growing synergistically with *E. coli* in low-nutrient conditions. Cooperative growth involved the exchange of soluble nutrients, including mucin- and carbohydrate-derived sugars and organic iron. Sialic acid was one such contributing sugar, consistent with previous reports^17,18^; however, a number of additional mono- and di-saccharides were produced by *Bacteroidales* in this nutritional context and supported *E. coli* growth. Human metagenomics analysis also supported the presence of carbohydrate cross-feeding, as multiple fiber- and sugar-degrading genes from *Bacteroides* spp. correlated with *E. coli* abundance in the microbiomes of stunted children.

Reciprocally, *E. coli* improved iron bioavailability for *Bacteroidales* in vitro, which might happen in a variety of ways: *Enterobacteriaceae* are known to secrete siderophores, degrade hemoglobin, and synthesize heme de novo^22,24^. Correlations between these taxa in the human microbiota was modulated by anemic status, suggesting that iron availability also plays an important role in cooperation in vivo. Furthermore, a *Bacteroides* spp. iron storage gene was one of the most strongly correlated with *Enterobacteriaceae* abundance in metagenomes from stunted children. Notably, iron usage by the microbiota has been shown to exacerbate anemia in mice, by directly competing with the host for iron uptake; the iron cross-feeding described here may therefore have additional consequences for host health^46^. It is also likely that other mechanisms not explored here, such as the exchange of amino acids and B vitamins, contribute to the synergistic growth advantage of these taxa in vitro and in vivo. Our gene-level analysis in humans was limited by the small number of available samples; further metagenomic sequencing efforts in populations of malnourished children would help to clarify some of these relationships.

Undernutrition is associated with significant microbial dysbiosis, but the factors driving this remain unclear; moreover, existing nutritional and sanitary interventions have failed to reverse child undernutrition. Both *Bacteroidetes* and *Enterobacteriaceae* have the potential to exacerbate EED and growth faltering in undernourished children, and have been shown to do so in mice. Here, we show that mutual growth synergy occurs between these taxa in protein- and iron-limited environments, underlying their co-occurrence in the gut microbiota of undernourished children. These data define a complex inter-species network with functional relevance to the undernourished gut and highlight a mechanism by which pathogen overgrowth might arise during undernutrition.

## Methods

### Bacterial strains and mutant construction

All bacterial strains, mutants, and plasmids used in this study are listed in Supplementary Table 2. *B. fragilis* wild-type strain 608R and ∆*feoAB* were a generous gift from Dr. Edson Rocha, East Carolina University^25^. *E. coli* Δ*nanA* and Δ*fucI* mutants were generated using homologous recombination to replace the target gene with a kanamycin resistance cassette (Kan^R^) following a protocol based on lambda red recombination^47^. Briefly, the Kan^R^ locus on pKD13 (Supplementary Table 2) was PCR-amplified using primers with 50 bp 5’ overhangs homologous to the target gene (Supplementary Table 7). Amplicons were purified by phenol chloroform extraction and electroporated into target *E. coli* carrying pkD46 expressing the lambda red recombinase. Transformants were selected on LB agar with 40 μg/mL kanamycin. Mutants were genotypically confirmed by PCR using primers flanking Kan^R^. Mutant phenotypes were confirmed by aerobic growth in M9 minimal media with fucose or sialic acid as a sole carbon source. Growth was assessed by OD_600_ using a plate reader (Tecan).

### Bacterial media and growth conditions

*Bacteroidales* and *E. coli* strains were routinely grown on Fastidious Anaerobic Agar (FAA) for 48 h at 37 °C and in Fastidious Anaerobic Broth (FAB) for 24 h at 37 °C with shaking at 250 rpm, under anaerobic conditions created by a sealed anaerobic jar and GasPak (BD). *E. coli* mutant strains were routinely grown with the addition of 40 µg/mL kanamycin and *B. fragilis* ∆*feoAB* was routinely grown with the addition of 10 µg/mL erythromycin.

Complex media for growth experiments were prepared according to the macronutrient quantities shown in Supplementary Table S1. In addition, all complex media contained, per 1 L: 13.6 g of KH_2_PO_4_; 0.875 g of NaCl; 1.125 g of (NH_4_)_2_SO_4_; 1.0 mL of histidine–hematin solution (1.9 mM hematin in 0.2M l-histidine); 1.0 mL MgCl_2_ solution (0.1 M in water); 1.0 mL vitamin K_3_ solution (1 mg/mL in ethanol); 1.0 mL CaCl_2_ solution (0.8% w/v in water); and 0.5 mL of vitamin B_12_ solution (0.01 mg/mL in water). Where specified, additional histidine, hematin or vitamin B12 was added to MAL-M media during preparation, to achieve tenfold the normal concentration (10 mL of histidine–hematin solution per L, and 5 mL of vitamin B12 solution). Media pH was adjusted to 7.2 with concentrated NaOH. Mucin was Type II Mucin from Porcine Stomach (PGM) (Sigma-Aldrich).

### *Bacteroidales*–*E. coli* community growth assays

Overnight cultures of *Bacteroidales* and *E. coli* in FAB were resuspended and washed twice in a carbon-free buffer (either PBS-/- or minimal media containing no carbon source) and normalized to OD_600_ = 0.35 in PBS. These normalized strains were then combined in equal volumes to prepare inoculation mixtures, with any missing strains replaced by the equivalent volume in PBS. Within an anaerobic chamber, 70 µL of bacterial inoculum was then added per 2 mL of degassed complex media, and co-cultures were grown anaerobically at 37 °C for 24 h with shaking. To assess growth outcomes, samples were serially diluted in PBS, plated, and grown on LB agar aerobically at 37 °C for 24 h to quantify *E. coli* growth, on FAA plates anaerobically to estimate total community growth, or on antibiotics-containing plates to enumerate mutant growth as appropriate. In the absence of a selective medium for all *Bacteroidales* species, *Bacteroidales* growth was quantified by qPCR. In all, 4–8 individual replicates are typically shown per growth assay, as stated in the figure legends for each panel. Each data point represents a single bacterial culture tube; further, each growth assay was repeated at least twice (i.e., from different seed cultures on a different day) with pooled results from all replicates shown. All media comparison experiments (Fig. 2 and Supplementary Fig. 2) were repeated at least three separate times with pooled results shown. The strain-wise replicates shown in Supplementary Fig. 3F–H (pooled in Supplementary Fig. 3D) represent a further level of replication, in that each “replicate” was performed by a different person using a different media batch; however each replicate still represents ≥8 bacterial tubes over at least two separate growth curve days.

### 16S rRNA qPCR to assess bacterial growth

The total abundance of *Bacteroidales* and *E. coli* was assessed using taxa-specific primers (Supplementary Table 7 and the Quantitect SYBR-Green Mastermix (Qiagen) following manufacturer’s protocols. Culture samples were heat-lysed at 100 °C for 15 min to release DNA. qPCR was performed on cell lysates diluted 1:10, using an Applied Biosystems^TM^ 7500 Fast Real-Time PCR Machine. C_T_ values were converted to 16S copy numbers using a standard curve generated from qPCR amplification of purified *B. thetaiotaomicron* DNA or purified *E. coli* DNA. Results were expressed in 16S copies/μL of culture.

### *E. coli* growth assessment in Bacteroidales supernatant

Endpoint cultures (24 h) of Bacteroidales or *E. coli* in MAL-M were centrifuged for 20 min at 16,000 × *g*, and the resultant supernatants were filter-sterilized through a 0.22-μm filter. Fresh overnight cultures of *E. coli* were then normalized to OD_600_ = 0.35 as described above and were subcultured 1:200 into a final volume of 200 μL of culture supernatant in a 96-well plate and grown anaerobically at 37 °C. OD_600_ was recorded every 15 min for 24 h using a plate reader (BioTek).

### Siderophore assay

Siderophore production was assayed via Chrome Azurol S (CAS) Plate Assay as previously reported^48^. Briefly, CAS agar plates for siderophore detection were prepared using acid-washed glassware according to the following recipe: 50 mL of CAS (30.25 mg/mL) was mixed with 10 mL of iron(III) solution (containing 160 µl of 6.25 M hydrochloric acid and 27.03 mg of FECl_3_.6H_2_O per 100 mL) and 40 mL of hexadecyltrimethyl ammonium bromide (1.82 mg/mL) and autoclaved. 100 mL of sterile CAS solution was then added to 900 mL freshly autoclaved minimal media (containing 1.0 g ammonium chloride, 1.704 g Na_2_HPO_4_, 1.089 g KH_2_PO_4_, 5.0 g sodium chloride, 30.24 g PIPES, 15 g agar, 1 mL of 1 M magnesium sulfate and 10 mL of 50% glucose). Overnight cultures of *E. coli* were grown on a low-iron succinate minimal media^49^. These Cultures were normalized to an O.D.600 of 0.3 and 3 μL of normalized culture were spotted onto a CAS indicator plate, for a total of nine spots per bacteria per plate. The negative control plate was 9 × 3 μL of the succinate minimal media. Plates were imaged after 24 h of incubation at 37 °C.

### Metabolomics analysis

Bacterial culture supernatants were obtained at 16 h and 24 h from 2 mL bacterial cultures as described above and were frozen at −70 °C prior to analysis. Low molecular weight sugars and NAc-sugar amines were quantified by LC-MRM/MS at The Metabolomics Innovation Centre (TMIC) commercial service (University of Victoria, Genome BC Proteomics Centre), according to previously published UPLC-MRM/MS methods^50^, with necessary modifications. In brief, a mixed stock solution of 13 low-MW sugars and 4 N-acetyl sugar amines was prepared with the use of their standard substances in 80% methanol at 500 µM for each compound. This solution was then serially diluted with the same solvent to prepare calibration solutions in a concentration range of 0.002 to 125 µM. For chemical derivatization, 20 µL of each medium sample or each calibration solution was mixed in turn with 20 µL of an internal standard solution containing ^13^C_6_-glucose, ^13^C_6_-mannose, ^13^C_6_-fructose, and ^13^C_5_-ribose in water, 40 µL of 200 mM 3-nitrophenylhydrazine hydrochloride solution in 60% methanol and 40 µL of 200 mM EDC.HCl solution prepared in a mixed solvent of methanol/water/pyridine (60:40:5, v/v/v). The mixture was allowed to react at 50 °C for 90 min. After the reaction, 20-µL aliquots were injected onto a Phenomenx PFP UPLC column (2.1 × 150 mm, 1.7 µm) to run LC-MS/MS on an Agilent 1290 UHPLC system coupled to an Agilent 6495B QQQ mass spectrometer, which was operated in the negative-ion multiple-reaction monitoring (MRM) mode. For LC separation, 0.1% formic acid in water/methanol (76/24, v/v) was used as the mobile phase at 0.25 mL/min and 40 °C for isocratic elution over a period of 15 min. The raw data were acquired using the Agilent MassHunter® 7.0 software. After data acquisitions, linearly regressed calibration curves of individual compounds were constructed with the analyte-to-internal standard peak area ratios measured from the injection of the calibration curves. For those compounds without their isotope-labeling analogs as the internal standards, ^13^C_6_-fructose was used a common internal standard. Concentrations of the analytes were calculated by interpolating the calibration curves of individual compounds with their analyte-to-internal standard peak area ratios measured from the injection of the sample solutions.

Quantification of short-chain fatty acids was performed in-house according to a method developed by Han et al., with minor modifications^51^. Briefly, 500 µL of supernatant were mixed with 500 µL of 50% acetonitrile, then the mixture was vortexed for 5 min and centrifuged at 7000 × *g* for 5 min. In total, 50 µL of the organic phase were derivatized adding 20 µL of 200 mM 3NPH in 50% acetonitrile and 20 µL 120 mM EDC solution of 6% pyridine in 50% acetonitrile. The mixture was left under agitation at 40 °C for 30 min. After this time reaction was stopped adding 100 µL of 0.1% formic acid in 90% acetonitrile. All samples were injected into the mass spectrometer right after being processed. An LC-MS/MS system that was equipped with an Agilent Technologies 1200 high-performance liquid chromatography (HPLC) instrument, an Agilent 6460 triple quadrupole (QQQ) mass spectrometer, and a reversed-phase column Zorbax 300 C18 250 × 4.6 mm (Agilent Technologies) was employed. MS was conducted in positive ion mode with an electrospray ionization voltage of 3500 V while using 50 psi nebulizer gas at a temperature of 350 °C. The sample injection volume was 5 µL. LC separation was performed while using mobile phase A (0.1% formic acid in 3% acetonitrile 97% water) and mobile phase B (0.1% formic acid in 90% acetonitrile), at a flow rate of 650 μL/min. The separation gradient was as follows: 20% B for 3 min., 20–30% B in 1 min. In all, 30–40% B in 7 min., 40–50% in 2 min., 50–100% in 2 min., 100% for 1 min., 100– 20 in 1 min., and 20% for 2 min. Collision energy of 10 V was used for multiple-reaction monitoring (MRM), and LC-MS/MS data were analyzed by Mass Hunter Qualitative Analysis B.06.00 software (Agilent Technologies). The identification and quantification of the SCFAs were carried out based on the retention time and mass fragmentation pattern compared with standards. Six-point calibration curves made by peak area vs concentration of the pure standards were used to quantify the different SCFA. The linearity of the curves was determined by the coefficient of determination (*R*^2^), being higher than 0.99 for all standards. Concentrations of the SCFAs were calculated by interpolating the calibration curves of individual compounds.

### High-throughput 16S sequencing for analysis of community growth kinetics

Culture samples were collected at 0, 16, and 24 h from bacteria grown in MAL-M as described above, and samples were heat-lysed at 100 °C for 15 min. The 16S rRNA gene (V4 region) was amplified directly from lysates using Illumina-tagged, barcoded primers 515F-806R^49^. Reactions were purified and normalized using the 96-well Sequal-Prep kit. Amplicons were pooled and gel-purified using a GeneJet Gel Extraction Kit (ThermoFisher) to remove primer dimers, and DNA yield was quantified by PicoGreen and qPCR. The final library was pooled and sequenced on an Illumina MiSeq platform with 30% PhiX, using the v2 kit for paired-end 250 bp reads.

### Bioinformatic analysis of in vitro bacterial community

Demultiplexed forward reads were analyzed in QIIME2^52^, using the DADA2 option for sequence quality control^53^ and trimming to 250 bp. Taxonomic assignment was performed using a Naive Bayes classifier trained on the SILVA database (v132, 99% OTU sequences, region 515F/806R)^54^. For *Bacteroides* which were not identified to the species level by the SILVA database, the 16S sequence was further queried using BLAST to rationally assign all five BG community members. Filtering at the amplicon sequence variant (ASV) level was performed in R using the phyloseq package^55^, to remove any ASVs that were not assigned to the initial community members (4.8% of all reads). Taxonomic barplots were then created using ggplot2^56^. To calculate the total abundance of each individual *Bacteroidales* species over time, relative abundances were multiplied by the total *Bacteroidales* abundance, as determined by 16S Bacteroidetes-specific qPCR, at each time point.

### Bioinformatic analysis of human fecal microbiota: 16S amplicon sequencing

16S rRNA gene sequencing of the V4 region were retrieved from four studies^11,12,28,57^ spanning five countries (Madagascar, Central African Republic, India, Malawi, and Peru) and included both publicly available data as well as new sequences available through the Afribiota study.

Children were included for analysis if they were 2–5 years of age and had linear growth measurements (height- or length-for-age- *z*-score) available as an outcome. Where longitudinal samples were available, a single sample at 2 years of age was included per child. For Dihn et al. 2016, height-for-age *z*-score was calculated from available height, age, and gender data using the public World Health Organization tool^58^. In all datasets, stunting was defined as HAZ ≤ −2 and nonstunted as HAZ > −2. To further bin samples by stunting, we defined stunting severity as follows: 3, “severely stunted” (HAZ ≤ −3); 2, “moderately stunted” (−3>HAZ ≤ −2); 1, “at risk of stunting” (−2>HAZ ≤ −1), and 0, “healthy” (HAZ > −1). Anemia was defined by a blood hemoglobin level of less than 11 g/100 mL after adjustment for altitude (hemoglobin levels in Madagascar were adjusted by −0.2 g/100 mL to account for the height above sea level, while hemoglobin levels in Central African Republic were approximately at sea level and were unadjusted)^59^.

Data from the Afribiota study were generated by co-authors Vonaesch PV and Sansonetti PJ^12^. The study protocol for Afribiota was approved by the Institutional Review Board of the Institut Pasteur (2016-06/IRB), the National Ethical Review Boards of Madagascar (55/MSANP/CE) and the Central African Republic (173/UB/FACSS/CSCVPER/16), and the Human Ethics Board of the University of British Columbia (H18-01108), in accordance with the principles of the Declaration of Helsinki. All participants received oral and written information about the study and the legal representatives of the children provided written consent to participate in the study^26^. The detailed inclusion and exclusion criteria and recruitment procedures of the Afribiota study are described in the published protocol^26^. Part of the dataset was previously published and is available at the European Nucleotide Archive (ENA) accession PRJEB27868. New sequencing data analyzed here are deposited at the ENA accession PRJEB48119.

To obtain new sequences through the Afribiota study, DNA was extracted with a QiaAmp cador^®^ Pathogen Mini kit using an additional bead-beating step and library preparation was performed as recommended by Kozich et al.^41^ using standard barcoded primers targeting the 16S V4 region (v4.SA501- v4.SA508 and v4.SA701- v4.SA712). The amplicon library was sequenced on a MiSeq using the MiSeq 500 Cycle V2 Reagent Kit (250 ×2). OTU table was generated using the Dada2 pipelines developed by the Holmes lab with slight modifications (https://benjjneb.github.io/dada2/^62^) and taxonomy was assigned using the Silva Database (version 132). The OTU table was transferred to Huus KE for further analysis.

Data from Desai et al. were obtained directly as an OTU table from their publicly available GitHub page (https://github.com/chandni177/BacterialViralMicrobiome_of_GrowthVelocity_in_EED)^27^. Raw sequencing data from Dinh et al.^28^ and Rouhani et al.^11^ were downloaded from the NCBI Sequence Read Archive (accession codes PRJNA279828 and PRJEB28159 respectively) using SRAtoolkit (v 2.10.8) and analyzed in qiime2 using the Deblur error correction option and SILVA taxonomic assignment (99% OTU sequences, region 515F/806R)^52,60^. Further processing of all studies was performed in R, including exclusion of taxa with low fractional abundance (<0.001%) and rarefaction to 5000 reads. Samples that were missing either taxon (i.e., zero values) were excluded for correlation analysis, and the relative abundance of nonzero values was log-transformed.

### Bioinformatic analysis of human fecal microbiota: shotgun sequencing

Shotgun metagenomics sequencing data of the human fecal microbiome were retrieved from two published studies in Zimbabwe and China^29,30^ that reported linear growth data in children. Children were included for analysis if they were 2–5 years of age and had linear growth measurements (HAZ) available as an outcome.

Raw sequencing data were retrieved from the NCBI Nucleotide Sequence Archive (accession codes PRJNA521455 and PRJNA543967) using SRAtoolkit (v 2.10.8) and processed according to the published Biobakery workflow (https://huttenhower.sph.harvard.edu/biobakery_workflows/)^61^. Sequence quality control and removal of human reads was performed using KneadData 0.7.4; bacterial abundances were calculated using MetaPhlan3.0; and bacterial gene abundances were calculated with Humann3 using nucleotide-based alignment against the Chocophlan database^62^. Gene abundance tables were grouped by uniref90_level4ec and normalized to copies per million (cpm) according to the standard pipeline.

Normalized taxa and gene abundance tables were imported into R. Taxonomic data were processed analogously to the 16S data using Phyloseq^55^, as described above and as detailed in the attached R markdown file. Also as described above, stunting was defined by HAZ, and samples that were missing either taxon (i.e., zero values) were excluded for correlation analysis. Species-level analysis was performed by pairwise Spearman’s correlation of all detected *Bacteroides* species against the abundance of *E. coli*, and associations were reported by raw *P* value for taxa that were consistent (positively or negatively associated) in both datasets individually. For subsequent gene-level analysis, genes from all *Bacteroides* species that were consistently positively associated with *E. coli* were binned together (i.e., summed per gene) and the gene table was further filtered for genes present in at least 60% (12 out of 20) samples, to try and reduce sparseness of the dataset while maintaining sample size. Within this subset of genes, pairwise Spearman’s correlations against the abundance of *Enterobacteriaceae* and *Escherichia* were performed, and both raw *P* value and FDR-corrected *P* value are reported. In the reciprocal analysis, annotated *E. coli* genes were filtered for genes present in at least 60% of samples and pairwise Spearman’s correlations were again performed against the abundance of *Bacteroidaceae* and *Bacteroides*.

### Summary figures

Summary figures (Figs. 1a and 6) were created with BioRender.com.

### Statistical analysis

Statistical analysis was performed in GraphPad Prism (www.graphpad.com) (v 9.1.1) or in R Studio (v 1.2.1335) using the packages phyloseq (v 1.32.0), ggplot2 (v 2.3.3.2), dplyr (v 1.0.2), tidyr (v 1.1.2), vegan (v 2.5.6), RColorBrewer (v 1.1.2), biomformat (v 1.16.0), reshape (v 2.1.4.4), and psych (v 2.0.8). Unless otherwise indicated, aggregate results represent the mean + /− SEM, and statistical significance is represented by *P value <0.05, **P value < 0.01, ***P value < 0.001 and ****P value < 0.0001. For in vitro experimental work, parametric tests were chosen based on the normal distribution of bacterial growth outcomes. Student’s two-sided *t* test was applied for comparisons of two groups; ANOVA for comparisons involving three or more groups; and two-way ANOVA for grouped analyses, for example, all analyses comparing single and co-culture across different media conditions. The post hoc test for ANOVA (Sidak, Tukey, or Dunnett) was chosen based on Prism’s default statistical recommendation, which takes into account the number of comparisons being tested. For human microbiota data analysis, nonparametric Spearman’s correlations were chosen based on the non-normal distribution of relative abundance data. Sample size, center and dispersion metrics, and statistical tests are reported in the figure legends. Exact *P* values and test statistics for all statistical results presented in the main and supplemental figures are listed in Supplementary Table 8.

### Reporting summary

Further information on research design is available in the Nature Research Reporting Summary linked to this article.

## Supplementary information

Supplementary Information

Reporting Summary

## Data Availability

New 16S rRNA sequencing data generated through the Afribiota project has been deposited to the ENA under accession PRJEB48119. Part of the Afribiota dataset was previously published and is available at the European Nucleotide Archive (ENA) accession PRJEB27868. All other sequencing data used in the study were previously published. Data from Desai et al.^27^ were obtained directly as an OTU table from their publicly available GitHub page (https://github.com/chandni177/BacterialViralMicrobiome_of_GrowthVelocity_in_EED). 16S rRNA sequencing data from Dinh et al.^28^ were retrieved from the NCBI Sequence Read Archive (SRA) under accession code PRJNA279828. 16S rRNA sequencing data from Rouhani et al.^11^ were retrieved from the NCBI SRA under accession code PRJEB28159. Shotgun metagenomics sequencing data from Osakunor et al.^30^ were retrieved from the NCBI SRA under accession code PRJNA521455. Shotgun metagenomics sequencing data from Li et al.^29^ were retrieved from the NCBI SRA under accession code PRJNA543967. The SILVA database v132 for 16S taxonomic assignment can be accessed from https://www.arb-silva.de/download/archive/qiime/. The Chocophlan database for metagenomics assignment was accessed from https://github.com/biobakery/humann#download-the-chocophlan-database. Raw metabolomics data have been deposited at Metabolomics Workbench accession number PR001175. Reagents and bacterial strains are available upon request. Source data are provided with this paper.

## Source data

Source Data

## Code availability

Code is available at https://github.com/khuus/Finlay_Public.
