# Supplementary material for: Cross-feeding between intestinal pathobionts promotes their overgrowth during undernutrition
Source: Nat Commun. 2021 Nov 25;12:6860. doi: 10.1038/s41467-021-27191-x (PMC8617199; doi:10.1038/s41467-021-27191-x)
Supplement: Supplementary file 1 — Supplementary Information [file 41467_2021_27191_MOESM1_ESM.pdf]

Supplementary Information

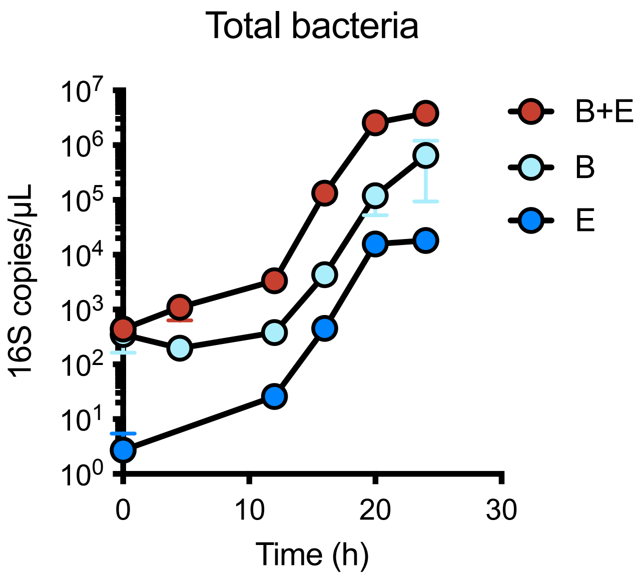

Supplementary Figure 1. Growth kinetics of *Bacteroidales* and *E. coli* in MAL-M. Total growth of co-culture, *Bacteroidales* and *E. coli* over time in MAL-M based on 16S rRNA gene qPCR (n=3). Mean +/- SEM. Source data are provided as a Source Data file.

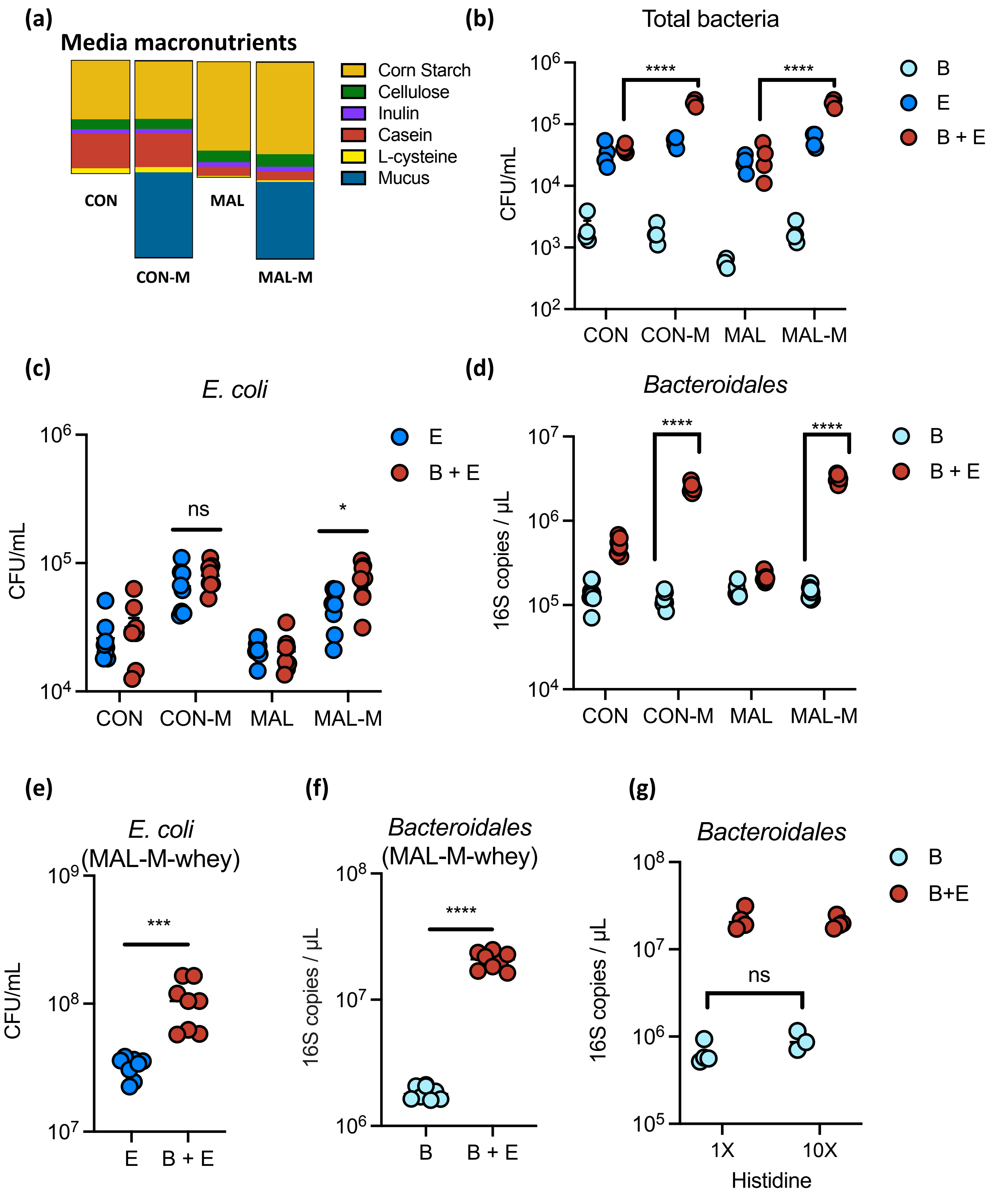

Supplementary Figure 2. Synergistic growth of *Bacteroidales* and *Enterobacteriaceae* is enhanced in mucin- and carbohydrate-rich complex media. (A) Media macronutrient compositions. After a 24h culture, bacterial endpoint growth was determined in these media for

(B) the total community (n=4), (C) *E. coli* (n=8) and (D) *Bacteroidales* (n=8). (E-F) Growth of (E) *E. coli* and (F) *Bacteroidales* in MAL-M media containing whey instead of casein as the protein source (n=8). (G) Growth of *Bacteroidales* in MAL-M media with 1X (0.2 mM) or 10X (2 mM) L-histidine (n=4). Mean +/- SEM are displayed. Significance was determined by two-way ANOVA with Tukey's post-hoc test (B-D, G) or by two-sided t test (E-F). **p* value <0.05, ***p* value <0.01 , ****p* value <0.001 and *****p* value <0.0001. B, *Bacteroidetes* mix; E*, E. coli* mix; CFU, colony forming units; Carb, carbohydrate medium; CarbM, carbohydrate plus mucus medium; Prot, protein medium; ProtM, protein plus mucus medium; MAL-M-whey, malnourished mucin medium plus whey. Source data are provided as a Source Data file. Exact p values and test statistics are reported in Supplementary Table 8.

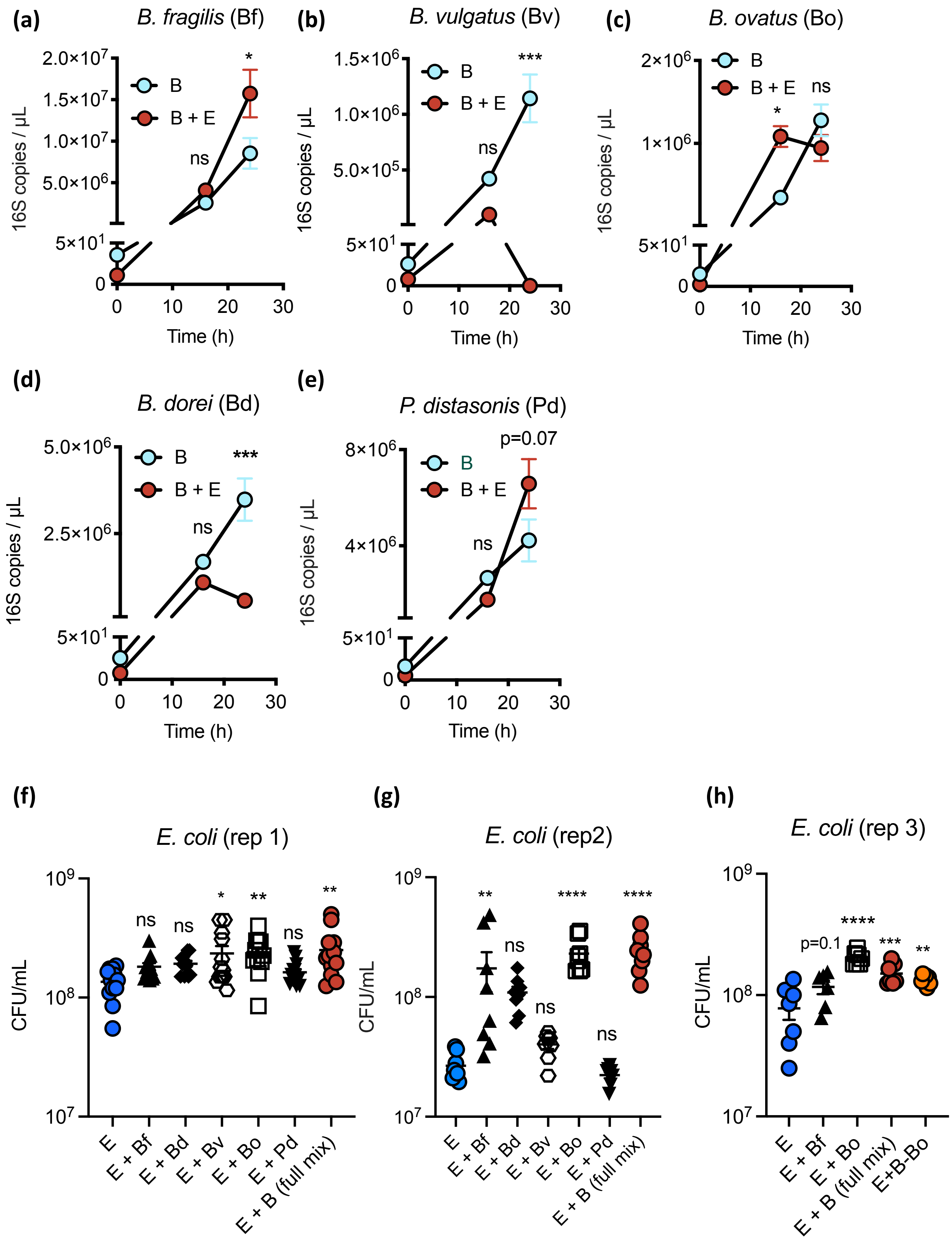

(a)
B. fragilis (Bf)
2.0×10^7
1.5×10^7
1.0×10^7
5.0×10^6
5×10^1
0
16S copies / µL
B
B + E
*
ns
0
10
20
30
Time (h)
(b)
B. vulgatus (Bv)
1.5×10^6
1.0×10^6
5.0×10^5
5×10^1
0
16S copies / µL
B
B + E
***
ns
Time (h)
(c)
B. ovatus (Bo)
2×10^6
1×10^6
5×10^1
0
16S copies / µL
B
B + E
ns
*
Time (h)
(d)
B. dorei (Bd)
5.0×10^6
2.5×10^6
5×10^1
0
16S copies / µL
B
B + E
***
ns
Time (h)
(e)
P. distasonis (Pd)
8×10^6
4×10^6
5×10^1
0
16S copies / µL
B
B + E
p=0.07
ns
Time (h)
(f)
E. coli (rep 1)
10^9
10^8
10^7
CFU/mL
ns
ns
*
**
ns
**
E
E + Bf
E + Bd
E + Bv
E + Bo
E + Pd
E + B (full mix)
(g)
E. coli (rep2)
10^9
10^8
10^7
CFU/mL
**
ns
ns
****
ns
****
E
E + Bf
E + Bd
E + Bv
E + Bo
E + Pd
E + B (full mix)
(h)
E. coli (rep 3)
10^9
10^8
10^7
CFU/mL
p=0.1
****
***
**
E
E + Bf
E + Bo
E + B (full mix)
E+B-Bo

Supplementary Figure 3. *Bacteroidales* strain-level kinetics. (A-E) Total growth over time of *B. fragilis* (A), *B. vulgatus* (B), *B. ovatus* (C), *B. dorei* (D) and *P. distasonis* (E) in MAL-M with or without *E. coli* (n=3). (F-H) Growth of *E. coli* with each individual *Bacteroidales* strain, separated by biological replicate (in this case, a biological replicate refers to a set of growth assays performed by the same person using the same media) (F) Replicate 1, n=12; (G) Replicate 2, n=8; (H) Replicate 3, n=7. In (H), *E. coli* was also grown in the full *Bacteroidales* mix minus *B. ovatus* (E+B-Bo) (n=7). Significance assessed by two-way ANOVA with post-hoc Sidak's test (A-E) or post-hoc Dunnett's test (F-H). **p* value <0.05, ***p* value <0.01 , ****p* value <0.001 and *****p* value <0.0001. Mean +/- SEM are displayed. Source data are provided as a Source Data file. Exact p values and test statistics are reported in Supplementary Table 8.

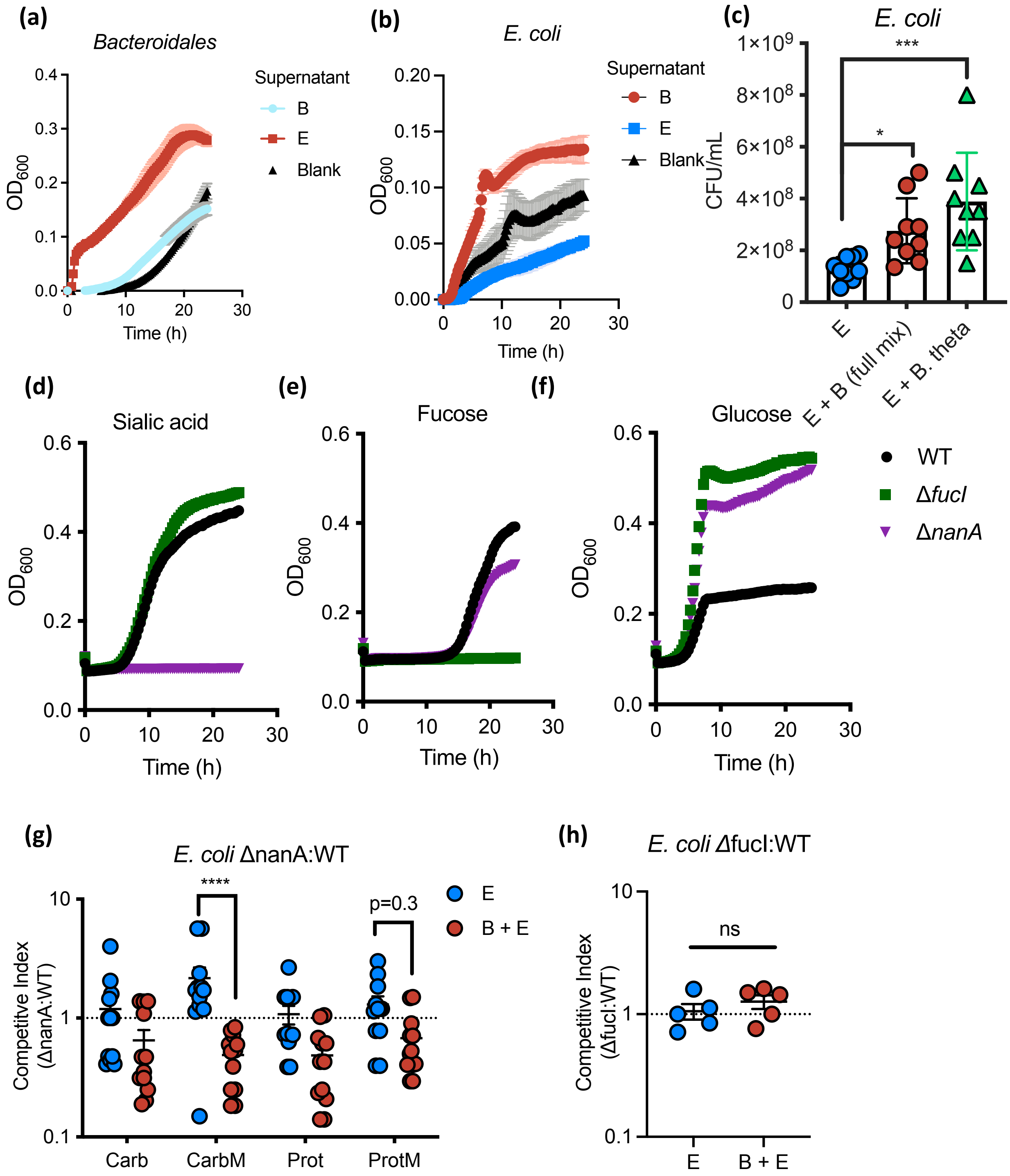

Supplementary Figure 4. Role of mucin-derived sugars in *E. coli* growth during co-culture. (A-B) Growth of *Bacteroidales* (A) and *E. coli* (B) over time (n=3), in the sterile filtered supernatants of blank MAL-M media ('Blank'), a 24h *E. coli* culture ('E'), or a 24h *Bacteroidales* culture ('B'). (C) Growth of *E. coli* at 24 endpoint with or without the *Bacteroidetes* mix or with *B. thetaiotaomicron* (B. theta) (n=9). (D-F) Growth of wild-type *E.*

coli , Δ*nanA E. coli* and Δ*fucI E. coli* in minimal M9 media with sialic acid (D), fucose (E) or glucose (F) as the sole carbon source (n=3). (G) Competitive index of Δ*nanA* versus WT *E. coli* in media with different macronutrient compositions (n=12). (H) Competitive index of Δ*fucI E. coli* compared to wild-type in the presence or absence of *Bacteroidetes,* in regular high-carbohydrate media (n=5). Mean +/- SEM are displayed. Significance was determined by ANOVA with post-hoc Dunnett's test (C), by two-way ANOVA with post-hoc Tukey's test (G) or by two-sided *t*-test (H). **p* value <0.05, ****p* value <0.001 and *****p* value <0.0001. B, *Bacteroidetes* mix; E*, E. coli* mix; CFU, colony forming units; Carb, carbohydrate media; CarbM, carbohydrate plus mucus media; Prot, protein media; ProtM, protein plus mucus media. Source data are provided as a Source Data file. Exact p values and test statistics are reported in Supplementary Table 8.

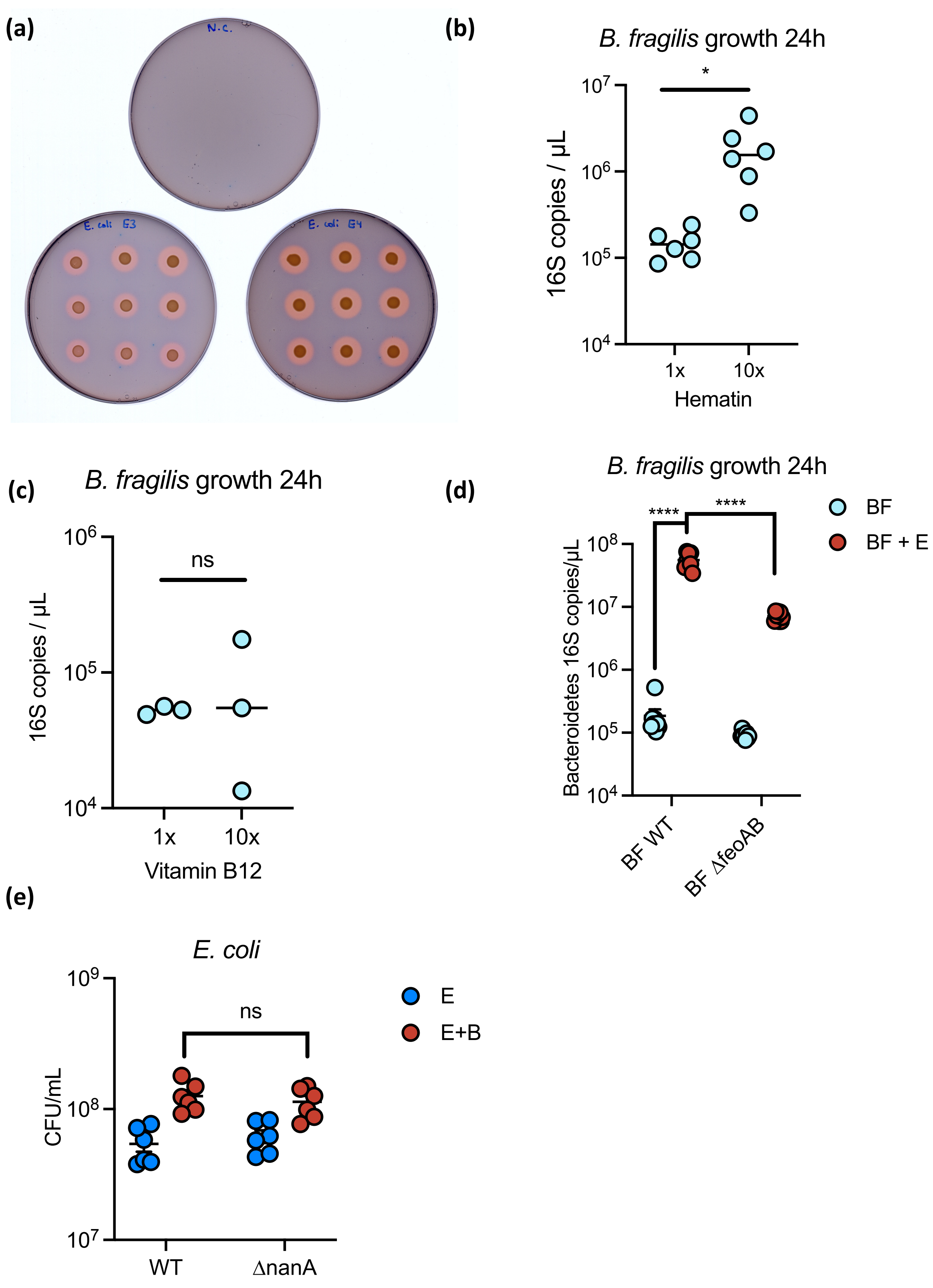
(a)
N.C.
E. coli E3
E. coli E4
(b)
B. fragilis growth 24h
*
16S copies / µL
10^7
10^6
10^5
10^4
1x
10x
Hematin
(c)
B. fragilis growth 24h
ns
16S copies / µL
10^6
10^5
10^4
1x
10x
Vitamin B12
(d)
B. fragilis growth 24h
****
****
BF
BF + E
Bacteroidetes 16S copies/µL
10^8
10^7
10^6
10^5
10^4
BF WT
BF ΔfeoAB
(e)
E. coli
ns
E
E+B
CFU/mL
10^9
10^8
10^7
WT
ΔnanA

Supplementary Figure 5. Metabolite production and growth dependency. (A) Siderophore production in *E. coli* strains 3_2_53 (EC3, bottom left) and 4_1_47 (EC4, bottom right), as visualized by an orange halo on CAS agar. Top plate, media-only negative control. (B) Growth of *B. fragilis* at 24h endpoint in MAL-M media with 1X (1.9 µM) or 10X (19 µM) hematin (n=6). (C) Growth of *B. fragilis* at 24h endpoint in media with 1X (0.004 µM) or 10X (0.04 µM) vitamin B12 (n=3). (D) Growth of WT strain 638R or Δ*feoAB B. fragilis* at 24h endpoint, in the presence or absence of *E. coli* (n=8). (E) Growth of WT or Δ*nanA E. coli* at 24h endpoint, in the presence or absence of *Bacteroidales* (n=6). Mean +/- SEM are displayed. Significance was determined by two-sided t test (B-C) and two-way ANOVA with post-hoc Tukey's test (D-E). **p* value <0.05, *****p* value <0.0001. BF, *B. fragilis*; E*, E. coli* mix. Source data are provided as a Source Data file. Exact p values and test statistics are reported in Supplementary Table 8.

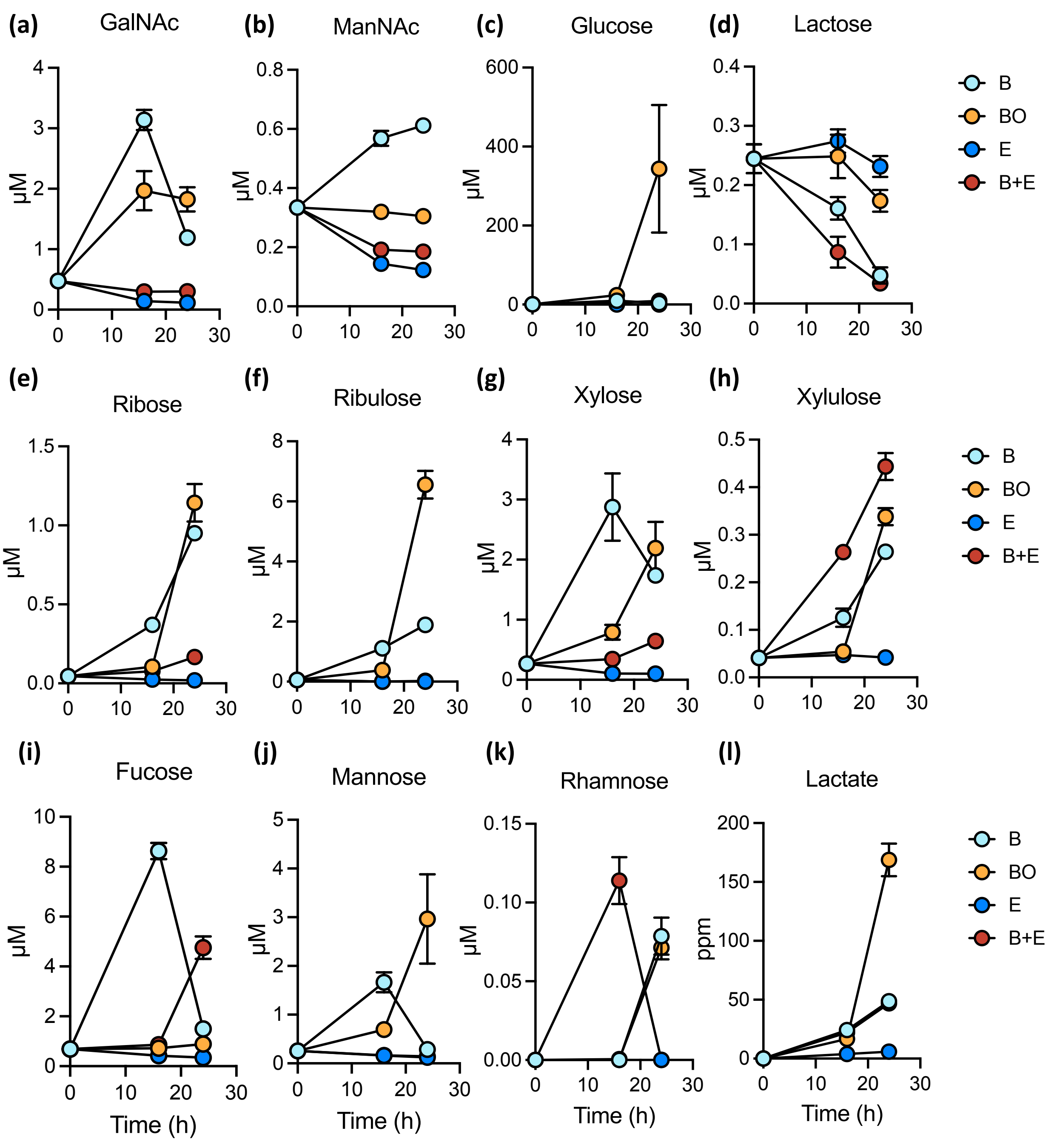

Supplementary Figure 6. Low-molecular weight sugars and short-chain fatty acids in *Bacteroidales* and *E. coli* cultures over time (n=3). Metabolites were quantified in sterile-filtered bacterial culture supernatants at 0, 16, and 24h. (A) N-acetylgalactosamine (GalNAc). (B) N-acetylmannosamine (ManNAc). (C) Glucose. (D) Lactose. (E) Ribose. (F) Ribulose. (G) Xylose. (H) Xylulose. (I) Fucose. (J) Mannose. (K) Rhamnose. (L) Lactate. B, *Bacteroidales* mix*;* BO, *B. ovatus;* E, *E. coli* mix*;* BE, *Bacteroidales-E. coli* co-culture. Mean +/- SEM are displayed. Source data are provided as a Source Data file.

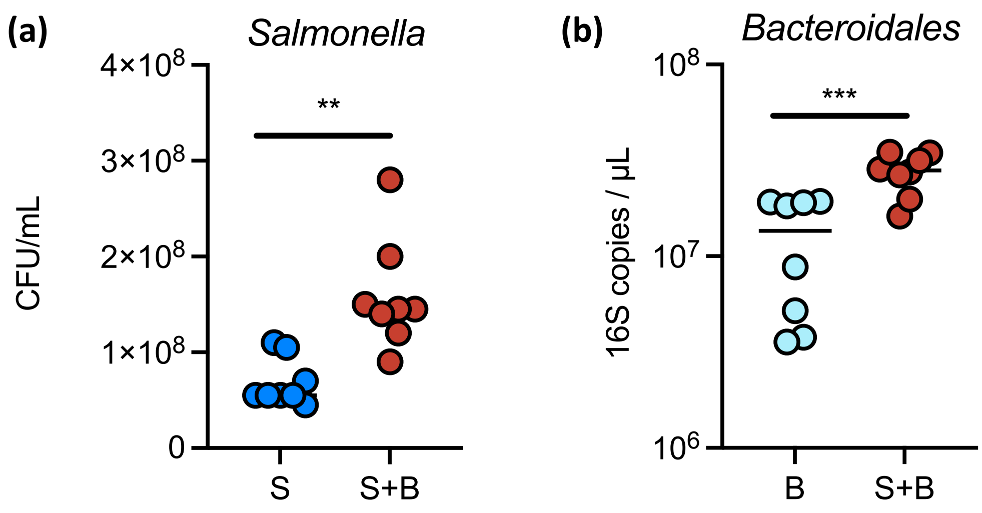

Supplementary Figure 7. Synergistic growth of *Bacteroidales* and *Salmonella.* (A) Growth of *Salmonella enterica* Typhimurium at 24h endpoint in the presence or absence of the five-strain *Bacteroidales* mix (n=8). (B) Growth of *Bacteroidales* in the presence or absence of *S. enterica* Typhimurium (n=8). Mean +/- SEM are displayed. Significance determined by two-sided *t*-test. ***p* value <0.01 , ****p* value <0.001. (A-B). Source data are provided as a Source Data file. Exact p values and test statistics are reported in Supplementary Table 8.

Supplementary Table 1. Macronutrient composition of complex media used in this paper.

Component	CON	CON-M	MAL	MAL-M	Prot	Prot-M	Carb	Carb-M
Corn Starch (g/L)	1.70	1.70	3.00	3.00	0.00	0.00	3.00	3.00
Cellulose (g/L)	0.30	0.30	0.40	0.40	0.00	0.00	0.4	0.40
Inulin (g/L)	0.12	0.12	0.15	0.15	0.00	0.00	0.15	0.15
Casein (g/L)	1.00	1.00	0.30	0.30	3.40	3.40	0.00	0.00
L-cysteine (g/L)	0.15	0.15	0.05	0.05	0.15	0.15	0.00	0.00
Mucus (g/L)	0.00	2.50	0.00	2.50	0.00	2.5	0.00	2.50

Supplementary Table 2. Bacterial strains and plasmids used in this paper.

Strain or Plasmid	Details	Reference
B. fragilis 3/1/12	Human fecal isolate	(1)
B. vulgatus 3/1/40A	Human fecal isolate	(1)
B. ovatus 3/8/47	Human fecal isolate	(1)
B. dorei 5/1/36	Human fecal isolate	(1)
P. distasonis 2/1/33B	Human fecal isolate	(1)
B. fragilis 638R WT	Clinical isolate, Rif^R	(2)
B. fragilis BER-51 ΔfeoAB	638R ΔfeoAB::tetQ, Rif^R Tet^R	(2)
B. thetaiotaomicron	Human fecal isolate	ATCC 29148
E. coli 3/1/53	Human fecal isolate	(1)
E. coli 4/1/47	Human fecal isolate	(1)
E. coli 3/1/53 ΔnanA	ΔnanA::kanR, Kan^R	This study
E. coli 4/1/47 ΔnanA	ΔnanA::kanR, Kan^R	This study
E. coli 3/1/53 ΔfucI	ΔfucI::kanR, Kan^R	This study
E. coli 4/1/47 ΔfucI	ΔfucI::kanR, Kan^R	This study
Salmonella enterica Typhimurium SL1344	Lab strain	(3)
pKD13	KanR	(4)
pkD46	Lambda red recombinase, AmpR	(4)

Supplementary Table 3. Summary of two-sided Spearman's correlations between *Bacteroidaceae* and *Enterobacteriaceae* log-adjusted relative abundance in shotgun metagenomics sequencing datasets, in samples where both taxa are present. Children were defined as either stunted (height-for-age z-score ≤-2) or non-stunted (height-for-age z-score >-2).

Dataset	Country	Nutritional Status	N	Rho	P
Li *et al.* 2019	China	All	36	0.02	0.8910
		Stunted	7	0.36	0.4444
		Non-stunted	29	0.03	0.8712
Osakunor *et al.* 2020	Zimbabwe	All	92	0.10	0.3197
		Stunted	13	0.59	**0.0360**
		Non-stunted	79	0.00	0.9681
Meta-analysis	All	All	128	0.05	0.5563
		Stunted	20	0.47	**0.0358**
		Non-stunted	108	-0.03	0.7196

Supplementary Table 4. Summary of species-level correlations between individual *Bacteroides* spp. and *E. coli* in human metagenomics datasets. Taxa are included in the table if they are consistent (in direction of correlation) in both datasets individually. Two-sided Spearman's rho and raw p-value are reported.

Species	Dataset	Rho	P
B. ovatus	Pooled	0.48	**0.0315**
	Li *et al.* 2019	0.60	0.1558
	Osakunor *et al.* 2020	0.53	0.0607
B. dorei	Pooled	0.44	**0.0489**
	Li *et al.* 2019	0.18	0.7011
	Osakunor *et al.* 2020	0.49	0.0920
B. uniformis	Pooled	0.44	0.0504
	Li *et al.* 2019	0.63	0.1289
	Osakunor *et al.* 2020	0.34	0.2547
B. finegoldii	Pooled	0.21	0.3791
	Li *et al.* 2019	0.20	0.6577
	Osakunor *et al.* 2020	0.23	0.7225
B. vulgatus	Pooled	0.19	0.4308
	Li *et al.* 2019	0.22	0.6414
	Osakunor *et al.* 2020	0.34	0.2547
B. plebeius	Pooled	0.69	0.97
	Li *et al.* 2019	0.26	0.5757
	Osakunor *et al.* 2020	0.21	0.4918
B. intestinalis	Pooled	-0.46	**0.0407**
	Li *et al.* 2019	-0.51	0.2369
	Osakunor *et al.* 2020	-0.48	0.1111
B. fragilis	Pooled	-0.30	0.2021
	Li *et al.* 2019	-0.25	0.5852
	Osakunor *et al.* 2020	-0.17	0.5655

Supplementary Table 5. Gene-abundance correlations in pooled human metagenomics datasets. *Bacteroides* spp. genes were correlated with *Enterobacteriaceae* abundance (N=20) and *E. coli* genes were correlated with *Bacteroidaceae* abundance (N=18 due to lack of detection of *E. coli* genes in two samples). Two-sided Spearman's rho and raw p-value are reported.

Taxon	EC Gene ID	Rho	P	Function
Bacteroides spp.	3.5.3.1: Arginase	0.52	0.0192	Amino acid metabolism
	1.4.3.5: Pyridoxal 5'-phosphate synthase	0.50	0.0255	Vitamin B6 synthesis
	1.16.3.2: Bacterial non-heme ferritin	0.46	0.0412	Iron storage
	4.2.3.3: Methylglyoxal synthase	0.46	0.0416	Glycolysis
	2.7.1.180: FAD:protein FMN transferase	0.45	0.0442	Generates flavoproteins
Escherichia	3.5.1.18: Succinyl-diaminopimelate desuccinylase	0.51	0.0317	Cell wall biosynthesis

Supplementary Table 6. Gene-abundance correlations in pooled human metagenomics datasets. *Bacteroides* spp. genes were correlated with *Escherichia* abundance (N=20). Two-sided Spearman's rho and raw p-value are reported. Functions of interest are highlighted by colour: yellow, carbohydrate metabolism; blue, B vitamin biosynthesis.

Bacteroides Gene	Spear_R	raw_P	Function
1.4.3.5: Pyridoxal 5'-phosphate synthase	0.62	0.0038	Vitamin B6 biosynthesis
3.4.13.20: Beta-Ala-His dipeptidase	0.59	0.0059	Peptidase
2.7.1.5: Rhamnulokinase	0.59	0.0060	Sugar (L-rhamnose) metabolism
1.7.2.2: Nitrite reductase (cytochrome ammonia-forming)	0.58	0.0073	Respiration
5.1.1.20: L-Ala-D/L-Glu epimerase	0.57	0.0093	Peptidoglycan recycling
4.1.99.19: 2-iminoacetate synthase	0.56	0.0101	Vitamin B1 biosynthesis
2.5.1.15: Dihydropteroate synthase	0.56	0.0106	Vitamin B9 biosynthesis
3.2.1.78: Mannan endo-1,4-beta-mannosidase	0.55	0.0111	Carbohydrate (mannan) degradation
3.4.24.55: Pitrilysin	0.55	0.0111	Peptidase
1.1.1.14: L-iditol 2-dehydrogenase	0.55	0.0125	Sugar alcohol metabolism
2.6.1.42: Branched-chain-amino-acid transaminase	0.55	0.0129	Amino acid metabolism
3.4.21.102: C-terminal processing peptidase	0.54	0.0137	Peptidase
4.2.1.17: Enoyl-CoA hydratase	0.54	0.0144	Fatty acid metabolism
3.6.4.13: RNA helicase	0.54	0.0144	Gene expression
4.1.2.19: Rhamnulose-1-phosphate aldolase	0.54	0.0150	Sugar (L-rhamnose) metabolism
2.1.1.191: 23S rRNA (cytosine(1962)-C(5))-methyltransferase	0.53	0.0165	rRNA methylation
4.3.1.17: L-serine ammonia-lyase	0.53	0.0168	Amino acid metabolism
2.7.8.8: CDP-diacylglycerol--serine O-phosphatidyltransferase	0.52	0.0184	Phospholipid biosynthesis
2.3.1.79: Maltose O-acetyltransferase	0.52	0.0188	Sugar (maltose) metabolism
5.4.2.6: Beta-phosphoglucomutase	0.52	0.0189	Glycolysis
3.5.2.6: Beta-lactamase	0.52	0.0197	Antibiotic resistance
3.6.1.22: NAD(+) diphosphatase	0.51	0.0202	NADH metabolism
2.4.2.53: Undecaprenyl-phosphate 4-deoxy-4-formamido-L-arabinose transferase	0.51	0.0203	Antibiotic resistance (modified lipid A)
3.2.1.135: Neopullulanase	0.51	0.0205	Carbohydrate (starch) degradation
3.2.1.4: Cellulase	0.51	0.0209	Carbohydrate (cellulose) degradation
1.1.1.18: Inositol 2-dehydrogenase	0.51	0.0220	Sugar alcohol metabolism
1.1.1.40: Malate dehydrogenase (oxaloacetate-decarboxylating) (NADP(+))	0.50	0.0232	Central metabolism
3.3.1.1: Adenosylhomocysteinase	0.50	0.0237	Amino acid biosynthesis
3.2.1.3: Glucan 1,4-alpha-glucosidase	0.49	0.0265	Carbohydrate (starch) degradation
3.2.1.41: Pullulanase	0.49	0.0271	Carbohydrate (starch) degradation
2.7.1.69: Protein-N(pi)-phosphohistidine--sugar phosphotransferase	0.49	0.0274	Sugar transport (PTS system)
3.2.1.89: Arabinogalactan endo-beta-1,4-galactanase	0.49	0.0293	Carbohydrate (arabinogalactan) degradation
1.1.1.103: L-threonine 3-dehydrogenase	0.48	0.0303	Amino acid metabolism
3.4.17.13: Muramoyltetrapeptide carboxypeptidase	0.48	0.0303	Peptidase
3.6.3.54: Cu(+) exporting ATPase	0.48	0.0305	Copper efflux
1.1.1.100: 3-oxoacyl-[acyl-carrier-protein] reductase	0.48	0.0320	Fatty acid biosynthesis
2.3.1.28: Chloramphenicol O-acetyltransferase	0.48	0.0321	Antibiotic resistance
3.2.1.31: Beta-glucuronidase	0.47	0.0387	Glycolysis
3.1.3.45: 3-deoxy-manno-octulosonate-8-phosphatase	0.46	0.0389	LPS biosynthesis
5.4.2.2: Phosphoglucomutase (alpha-D-glucose-1,6-bisphosphate-dependent)	0.46	0.0396	Glycolysis
3.1.3.27: Phosphatidylglycerophosphatase	0.46	0.0407	Phospholipid biosynthesis
1.6.5.3: NADH:ubiquinone reductase (H(+)-translocating)	0.46	0.0409	Respiration
6.1.1.2: Tryptophan--tRNA ligase	0.46	0.0426	Translation
3.1.4.46: Glycerophosphodiester phosphodiesterase	0.45	0.0447	Phospholipid metabolism
2.4.1.320: 1 4-beta-mannosyl-N-acetylglucosamine phosphorylase	0.45	0.0463	Carbohydrate degradation
1.10.3.10: Ubiquinol oxidase (H(+)-transporting)	0.45	0.0465	Respiration
1.17.4.2: Ribonucleoside-triphosphate reductase	0.44	0.0496	DNA synthesis and repair

Supplementary Table 7. Primers used in this paper.

Primers	Application	Sequence	Reference
Bacteroidetes_16S_F<br>Bacteroidetes_16S_R	qPCR	GGTTCTGAGAGGAAGGTCCC<br>GCTGCCTCCCGTAGGAGT	(1)
Enterobacteriaceae_16S_F<br>Enterobacteriaceae_16S_R	qPCR	CATTGACGTTACCCGCAGAAGAAGC<br>CTCTACGAGACTCAAGCTTGC	(1)
Eubacteria_16S_F<br>Eubacteria_16S_R	qPCR	ACTCCTACGGGAGGCAGCAGT<br>ATTACCGCGGCTGCTGGC	(1)
ΔnanA_P1	Cloning	ATAAAGGTATATCGTTTTATCAGACAAGCATCACT<br>TCAGAGGTATTTATGATTCCGGGGATCCGTCGAC<br>C	This paper
ΔnanA_P2		CCCGGTAGGGGCGAGCGAGGGGAAACAACTCAC<br>CCGCGCTCTTGCATCAATGTAGGCTGGAGCTGCTT<br>CG	
ΔfucI_P1	Cloning	ACGGCAACTAACTGAACATATTTTCCGAATAAAGT<br>GAGGAATCTGTAATGATTCCGGGGATCCGTCGAC<br>C	This paper
ΔfucI_P2	Cloning	GACCGGGCATCACATCAGGGAGTAATGTATTAAC<br>GCTTGTACAACGGGCCTGTAGGCTGGAGCTGCTT<br>CG	
Kan_k1<br>Kan_k2	Cloning	CAGTCATAGCCGAATAGCCT<br>CGGTGCCCTGAATGAACTGC	(4)
V4 Index Primers i5	Sequencing	AATGATACGGCGACCACCGAGATCTACAC[index]<br>TA TGGTAATTGTGTGCCAGCMGCCGCGGTAA	(5)
V4 Index Primers i7	Sequencing	CAAGCAGAAGACGGCATACGAGAT[index]AGTC<br>AGTCAG CCGGACTACHVGGGTWTCTAA T	

Supplementary Table 8. Exact p values and test statistics for statistical results displayed in the Main and Supplementary Figures. Note that GraphPad PRISM was used for most statistical tests, as described in the Methods, and does not report exact p values beyond 4 significant digits (e.g. below <0.0001).

Figure	Panel	Comparison	Test	DF	P	test statistic	95% confidence
1	C	E vs B+E	two-sided t test	14	0.0008	4.239	100973267 to 307776733
1	D	B vs B+E	two-sided t test	14	<0.0001	24.13	2779447 to 3321689
2	B	CarbM(B+E) vs ProtM(B+E)	post-hoc Tukey's test	71	0.0086	5.621	46138851 to 570111149
2	C	CarbM(E) vs CarbM(B+E)	post-hoc Tukey's test	69	<0.0001	9.388	-163928084 to -58971916
2	C	CarbM(B+E) vs ProtM(B+E)	post-hoc Tukey's test	69	<0.0001	9.456	59771916 to 164728084
2	D	CarbM(B) vs CarbM(B+E)	post-hoc Tukey's test	56	<0.0001	10.79	-29374636 to -12212423
2	D	CarbM(B+E) vs ProtM(B+E)	post-hoc Tukey's test	56	<0.0001	12.16	10049841 to 27212054
2	E	1X(E) vs 1X(B+E)	post-hoc Tukey's test	27	<0.0001	15.72	-154767198 to -93625659
2	E	1X(E) vs 10X(E)	post-hoc Tukey's test	27	0.7909	1.31	-39534169 to 19534169
2	E	1X(B+E) vs 10X(B+E)	post-hoc Tukey's test	27	0.0017	5.832	15500659 to 76642198
2	F	1X(B) vs 1X(B+E)	post-hoc Tukey's test	28	<0.0001	17.24	-23369473 to -14816715
2	F	1X(B) vs 10X (B)	post-hoc Tukey's test	28	<0.0001	14.83	-20700460 to -12147702
2	F	1X(B+E) vs 10X(B+E)	post-hoc Tukey's test	28	0.0030	2.41	1806565 to 10359323
3	A	BF (B vs B+E)	post-hoc Sidak's test	20	<0.0001	10.81	-0.1456 to -0.08507
3	A	BD (B vs B+E)	post-hoc Sidak's test	20	<0.0001	14.68	0.1264 to 0.1869
3	A	BV (B vs B+E)	post-hoc Sidak's test	20	0.0001	5.404	0.02740 to 0.08793
3	A	BO (B vs B+E)	post-hoc Sidak's test	20	0.0068	3.717	0.009403 to 0.06993
3	A	PD (B vs B+E)	post-hoc Sidak's test	20	0.6393	1.374	-0.04493 to 0.01560
3	B	0h (B vs B+E)	post-hoc Sidak's test	12	0.0185	3.313	-0.1745 to -0.01555
3	B	24h (B vs B+E)	post-hoc Sidak's test	12	<0.0001	11.06	0.2378 to 0.3967
3	C	BF (B vs B+E)	two-sided t test (fdr correction)	28	4.11E-05	6.025	14446552 to 29331692
3	C	BD (B vs B+E)	two-sided t test (fdr correction)	14	4.70E-05	8.81	1446914 to 2378051

3	C	BV (B vs B+E)	two-sided t test (fdr correction)	14	2.07E-05	11.5	1826235 to 2663287
3	C	BO (B vs B+E)	two-sided t test (fdr correction)	28	3.53E-06	8.449	12677886 to 20792775
3	C	PD (B vs B+E)	two-sided t test (fdr correction)	14	4.70E-05	8.853	1017235 to 1667724
3	D	E vs E+BF	post-hoc Dunnett's test	159	0.0122	3.102	-139487293 to -12109573
3	D	E vs E+BD	post-hoc Dunnett's test	159	0.0527	2.587	-138281086 to 536447
3	D	E vs E+BV	post-hoc Dunnett's test	159	0.048	2.622	-137173425 to -408057
3	D	E vs E+BO	post-hoc Dunnett's test	159	<0.0001	5.757	-202418494 to -76248173
3	D	E vs E+PD	post-hoc Dunnett's test	159	0.9616	0.7011	-86773425 to 49991943
3	D	E vs E+B (full mix)	post-hoc Dunnett's test	159	<0.0001	5.559	-197603679 to -71433358
4	A	E vs B	post-hoc Dunnett's test	18	0.0001	8.998	0.05078 to 0.08770
4	B	B vs E	post-hoc Dunnett's test	9	0.0001	10.33	-0.1750 to -0.07818
4	C	E vs B+E	two-sided t test	10	0.0008	4.7	-3.253 to -1.161
4	D	BF vs BF+E	two-sided t test	10	0.0180	2.827	-1.555 to -0.1841
4	L	E vs E+ NeuNAc	post-hoc Dunnett's test	20	0.0368	2.687	-188052752 to -5280581
4	L	E vs E + fructose	post-hoc Dunnett's test	20	0.0274	2.826	-193052752 to -10280581
4	L	E vs E + maltose	post-hoc Dunnett's test	20	<0.0001	6.394	-321386086 to -138613914
S2	B	CON(B+E) vs CONM(B+E)	post-hoc Tukey's test	36	<0.0001	25.78	-213867 to -145133
S2	B	MAL(B+E) vs MALM(B+E)	post-hoc Tukey's test	36	<0.0001	27.06	-222742 to -154008
S2	C	CONM(E) vs CONM(B+E)	post-hoc Tukey's test	56	0.7214	2.326	-40614 to 12739
S2	C	MALM(E) vs MALM(B+E)	post-hoc Tukey's test	56	0.0489	4.465	-53426 to -73.72
S2	D	CONM(B) vs CONM(B+E)	post-hoc Tukey's test	55	<0.0001	26.38	-2619859 to -2041099
S2	D	MALM(B) vs MALM(B+E)	post-hoc Tukey's test	55	<0.0001	35.67	-3324269 to -2765133
S2	E	E vs B+E	two-sided t test	14	0.0004	4.595	38661905 to 106338095

S2	F	B vs B+E	two-sided t test	14	<0.0001	16.39	16305976 to 21216524
S2	G	1X (B) vs 10X (B)	post-hoc Tukey's test	11	0.9997	0.1314	-8746618 to 8222830
S3	A	BF (B vs B+E) 24h	post-hoc Sidak's test	8	0.0353	2.972	-13854771 to -555059
S3	B	BV (B vs B+E) 24h	post-hoc Sidak's test	8	0.0001	7.478	724623 to 1564336
S3	C	BO (B vs B+E) 16h	post-hoc Sidak's test	8	0.0112	3.749	-1278234 to -197956
S3	C	BO (B vs B+E) 24h	post-hoc Sidak's test	8	0.2412	1.693	-206809 to 873470
S3	D	BD (B vs B+E) 24h	post-hoc Sidak's test	8	0.0004	6.543	1693322 to 4138373
S3	E	PD (B vs B+E) 24h	post-hoc Sidak's test	8	0.0773	2.458	-4985062 to 273206
S3	F	E vs E+BF	post-hoc Dunnett's test	76	0.5002	1.46	-129447249 to 36947249
S3	F	E vs E+BD	post-hoc Dunnett's test	76	0.3162	1.756	-141923144 to 28211023
S3	F	E vs E+BV	post-hoc Dunnett's test	76	0.0124	3.144	-182780582 to -16386084
S3	F	E vs E+BO	post-hoc Dunnett's test	76	0.0078	3.302	-187780582 to -21386084
S3	F	E vs E+PD	post-hoc Dunnett's test	76	0.8938	0.8683	-110697249 to 55697249
S3	F	E vs E+B (full mix)	post-hoc Dunnett's test	76	0.0028	3.631	-198197249 to -31802751
S3	G	E vs E+BF	post-hoc Dunnett's test	49	0.0045	3.556	-256143551 to -36981449
S3	G	E vs E+BD	post-hoc Dunnett's test	49	0.2093	1.994	-191768551 to 27393551
S3	G	E vs E+BV	post-hoc Dunnett's test	49	0.9995	0.3276	-123081051 to 96081051
S3	G	E vs E+BO	post-hoc Dunnett's test	49	<0.0001	4.95	-313581051 to -94418949
S3	G	E vs E+PD	post-hoc Dunnett's test	49	0.9999	0.1062	-105206051 to 113956051
S3	G	E vs E+B (full mix)	post-hoc Dunnett's test	49	<0.0001	5.284	-327331051 to -108168949
S3	F	E vs E+B (full mix)	post-hoc Dunnett's test	29	0.0004	4.466	-115041938 to -30672348
S3	F	E vs E+BF	post-hoc Dunnett's test	29	0.0951	2.286	-82716850 to 5097803
S3	F	E vs E+BO	post-hoc Dunnett's test	29	<0.0001	7.662	-167184795 to -82815205

S3	F	E vs E + B - BO	post-hoc Dunnett's test	29	0.0061	3.459	-98613366 to -14243777
S4	C	E vs E+B (full mix)	post-hoc Dunnett's test	31	0.0361	2.616	-284163888 to -8058335
S4	C	E vs E + B.theta	post-hoc Dunnett's test	31	0.0002	4.645	-397497221 to -121391668
S4	G	CarbM(E) vs CarbM(B+E)	post-hoc Sidak's test	88	<0.0001	4.893	0.8059 to 2.550
S4	G	ProtM(E) vs ProtM(B+E)	post-hoc Sidak's test	88	0.2634	1.811	-0.2510 to 1.493
S4	H	E vs B+E	two-sided t test	8	0.3807	0.9277	-0.3079 to 0.7224
S5	B	1x:BF vs. 10x:BF	two-sided t test	10	0.0158	2.902	396960 to 3022281
S5	C	1x:BF vs. 10x:BF	two-sided t test	4	0.59	0.5849	-106717 to 163680
S5	D	BF vs BF + E (WT BF)	post-hoc Tukey's test	28	<0.0001	19.91	-65898627 to -44491617
S5	D	BF+E (WT) vs BF+E (ΔfeoAB)	post-hoc Tukey's test	28	<0.0001	17.41	37546813 to 58953823
S5	E	WT(E+B) vs ΔnanA(E+B)	post-hoc Tukey's test	20	0.8416	1.168	-28975906 to 53225906
S7	A	S vs S+B	two-sided t test	14	0.0012	4.041	42234192 to 137765808
S7	B	B vs S+B	two-sided t test	14	0.0007	4.337	7738129 to 22877662

Supplementary References

1. Brown EM, Wlodarska M, Willing BP, Vonaesch P, Han J, Reynolds LA, Arrieta M-C, Uhrig M, Scholz R, Partida O, Borchers CH, Sansonetti PJ, Finlay BB. 2015. Diet and specific microbial exposure trigger features of environmental enteropathy in a novel murine model. Nature Communications 6:7806.
2. Rocha ER, Bergonia HA, Gerdes S, Jeffrey Smith C. 2019. Bacteroides fragilis requires the ferrous-iron transporter FeoAB and the CobN-like proteins BtuS1 and BtuS2 for assimilation of iron released from heme. Microbiologyopen 8:e00669.
3. Ferreira RBR, Valdez Y, Coombes BK, Sad S, Gouw JW, Brown EM, Li Y, Grassl GA, Antunes LCM, Gill N, Truong M, Scholz R, Reynolds LA, Krishnan L, Zafer AA, Sal-Man N, Lowden MJ, Auweter SD, Foster LJ, Finlay BB. 2015. A Highly Effective Component Vaccine against Nontyphoidal *Salmonella enterica* Infections. mBio 6:e01421-15.
4. Datsenko KA, Wanner BL. 2000. One-step inactivation of chromosomal genes in Escherichia coli K-12 using PCR products. PNAS 97:6640–6645.
5. Kozich JJ, Westcott SL, Baxter NT, Highlander SK, Schloss PD. 2013. Development of a Dual-Index Sequencing Strategy and Curation Pipeline for Analyzing Amplicon Sequence Data on the MiSeq Illumina Sequencing Platform. Applied and Environmental Microbiology 79:5112–5120.
