# Supplementary material for: Cross-feeding between intestinal pathobionts promotes their overgrowth during undernutrition
Source: Nat Commun. 2021 Nov 25;12:6860. doi: 10.1038/s41467-021-27191-x (PMC8617199; doi:10.1038/s41467-021-27191-x)
Supplement: Supplementary file 2 — Reporting Summary [file 41467_2021_27191_MOESM2_ESM.pdf]

nature portfolio

Corresponding author(s): B. Brett Finlay

Last updated by author(s): Oct 28, 2021

Reporting Summary

Nature Portfolio wishes to improve the reproducibility of the work that we publish. This form provides structure for consistency and transparency in reporting. For further information on Nature Portfolio policies, see our Editorial Policies and the Editorial Policy Checklist.

Statistics

For all statistical analyses, confirm that the following items are present in the figure legend, table legend, main text, or Methods section.

n/a	Confirmed	
☐	☒	The exact sample size (*n*) for each experimental group/condition, given as a discrete number and unit of measurement
☐	☒	A statement on whether measurements were taken from distinct samples or whether the same sample was measured repeatedly
☐	☒	The statistical test(s) used AND whether they are one- or two-sided<br>*Only common tests should be described solely by name; describe more complex techniques in the Methods section.*
☐	☒	A description of all covariates tested
☐	☒	A description of any assumptions or corrections, such as tests of normality and adjustment for multiple comparisons
☐	☒	A full description of the statistical parameters including central tendency (e.g. means) or other basic estimates (e.g. regression coefficient) AND variation (e.g. standard deviation) or associated estimates of uncertainty (e.g. confidence intervals)
☐	☒	For null hypothesis testing, the test statistic (e.g. *F*, *t*, *r*) with confidence intervals, effect sizes, degrees of freedom and *P* value noted<br>*Give P values as exact values whenever suitable.*
☒	☐	For Bayesian analysis, information on the choice of priors and Markov chain Monte Carlo settings
☒	☐	For hierarchical and complex designs, identification of the appropriate level for tests and full reporting of outcomes
☐	☒	Estimates of effect sizes (e.g. Cohen's *d*, Pearson's *r*), indicating how they were calculated

Our web collection on statistics for biologists contains articles on many of the points above.

Software and code

Policy information about availability of computer code

Data collection	To download and process 16S and metagenomic sequencing data from the NCBI SRA: qiime2-2020.8; sratoolkit.2.10.8-centos_linux64; python=3.7; kneaddata v0.7.4; metaphlan=3.0=phy5ca1d4c_4; humann3
Data analysis	To analyze data in R: Rstudio 1.2.1335; R 4.0.2; phyloseq 1.32.0; ggplot2.3.3.2; dplyr 1.0.2; tidyr 1.1.2; vegan 2.5.6; RColorBrewer 1.1.2; biomformat 1.16.0; reshape2.1.4.4; psych 2.0.8; GraphPad Prism 9.1.1. R Code for analysis has been made available on GitHub: https://github.com/khuus/Finlay_Public.

For manuscripts utilizing custom algorithms or software that are central to the research but not yet described in published literature, software must be made available to editors and reviewers. We strongly encourage code deposition in a community repository (e.g. GitHub). See the Nature Portfolio guidelines for submitting code & software for further information.

Data

Policy information about availability of data

All manuscripts must include a data availability statement. This statement should provide the following information, where applicable:

- Accession codes, unique identifiers, or web links for publicly available datasets
- A description of any restrictions on data availability
- For clinical datasets or third party data, please ensure that the statement adheres to our policy

New 16S rRNA sequencing data generated through the Afribiota project has been deposited to the ENA under accession PRJEB48119. All other sequencing data used in the study were previously published. Data from Desai et al. were obtained directly as an OTU table from their publicly available GitHub page (https://github.com/chandni177/BacterialViralMicrobiome_of_GrowthVelocity_in_EED). 16S rRNA sequencing data from Dinh et al. were retrieved from the NCBI Sequence Read Archive (SRA) under accession code PRJNA279828 [https://www.ncbi.nlm.nih.gov/bioproject/PRJNA279828]. 16S rRNA sequencing data from Rouhani et al.

were retrieved from the NCBI SRA under accession code PRJEB28159 [https://www.ncbi.nlm.nih.gov/bioproject/?term=PRJEB28159]. Shotgun metagenomics sequencing data from Osakunor et al. were retrieved from the NCBI SRA under accession code PRJNA521455 [https://www.ncbi.nlm.nih.gov/bioproject/?term=PRJNA521455]. Shotgun metagenomics sequencing data from Li et al. were retrieved from the NCBI SRA under accession code PRJNA543967 [https://www.ncbi.nlm.nih.gov/bioproject/?term=PRJNA543967]. The SILVA database v132 for 16S taxonomic assignment can be accessed here: https://www.arb-silva.de/download/archive/qiime/. The Chocophlan database for metagenomics assignment was accessed here: https://github.com/biobakery/humann#download-the-chocophlan-database.
Raw metabolomics data have been deposited at Metabolomics Workbench accession number PR001175 [http://dx.doi.org/10.21228/M8TM5F].
Source data are provided with this paper. Reagents and bacterial strains are available upon request.

Field-specific reporting

Please select the one below that is the best fit for your research. If you are not sure, read the appropriate sections before making your selection.

☒ Life sciences ☐ Behavioural & social sciences ☐ Ecological, evolutionary & environmental sciences

For a reference copy of the document with all sections, see nature.com/documents/nr-reporting-summary-flat.pdf

Life sciences study design

All studies must disclose on these points even when the disclosure is negative.

Sample size

No statistical sample size calculations were performed in this study. For in vitro bacterial growth experiments, sample sizes were chosen based on common good practise (at least 3 replicates) and practical considerations (number of tubes that could be processed at once). For in vitro growth experiments shown in Fig 1-4 and in Fig S1-S7 we therefore normally had n=6-8 per condition, based on a sample size of 3-4 biological replicates per condition on a single day and the experiment repeated independently at least twice. These sample sizes are sufficient based on the large effect size and relatively low variance observed.

In Fig S3F-H and in Fig 3D, sample size was increased because we observed relatively high variance and smaller effect sizes between strains.

In Fig 3A (16S rRNA sequencing) and in Fig 4E-K (metabolite quantification) we included only 3 samples per condition, as a practical and cost consideration based on the higher investment of performing those experiments. In Fig S4D-F, only 3 samples per condition were performed for the mutant growth curves because this confirmed expected mutant phenotypes with a large effect (presence/absence of metabolism).

For the human microbiome analysis in Fig 5 and Table 1, sample size was based on the number of samples available for analysis that fit our inclusion criteria. This sample size was sufficient given the consistent and significant trends in each dataset alone and in the pooled analysis (see Table 1).

Data exclusions

Data were not excluded from in vitro analyses, unless the culture was clearly contaminated by the wrong bacterium (i.e. by aerobic plating or by qPCR of the output, or by sanger sequencing of the input) or accurate CFU counting was not possible for the sample (e.g. smeared colonies due to condensation dripping onto the plate). Samples were excluded from the human microbiota analysis as defined in the methods, based on age criteria and if either taxon of interest was missing in the sample.

Replication

Our main findings of Bacteroides-E. coli synergy were reproduced countless times (estimate: 20 times shown throughout the figures and at least as many other times not shown). We had three independent experimentalists perform the central growth experiments of the paper over the course of its research, and achieved comparable results each time. All attempts at replicating this finding (using uncontaminated cultures and the correct medium) were successful.

Replication was also successful for all the media-based preparations in Fig 2 and Fig S2. Panels 2B-D and S2B-D each represent pooled data from four independent replicates (different experimental days), each of which showed similar trends. In addition, these data are a reproduction of previous results with very slightly different media compositions by a different experimentalist, which are not included in the paper, but which also replicated successfully.

The 16S relative abundance analysis of different Bacteroides in Fig 3A was only performed once independently (on three biological samples). However, we further expanded on those results by testing individual strain-level contributions experimentally (Fig 3C-D, Fig S3F-H), and these experiments were repeated >8 times. There was some variability in between-strain differences between replicates, possible due to media or timing batch effects, which is acknowledged in the text and in the supplemental figures.

The mutant experiments shown in Figure 4 and S4 for ΔnanA replicated at least 6 times (twice for the competition experiment in regular IMM and four times across the different media preparations in S4G) and for ΔfeoAB replicated at least 4 times (twice as a competition and twice as an individual growth phenotype).

The metabolite quantification shown in Figure 4 and Fig S6 was only performed once independently (on three biological replicates however) because of the expense of the experiment. However, metabolite patterns largely confirmed previous findings on sugars exchanged and released by Bacteroidetes spp, suggesting these findings are reproducible between research groups.

Our microbiome analyses in silico, were replicated in multiple independently collected human datasets. All available datasets which fit our inclusion criteria (age and stunting phenotypes) replicated successfully except for the smallest 16S dataset (N=8 stunted children) and all are listed in Table 1. Both metagenomics datasets were analyzed during revisions and also replicated this finding. Two further metagenomics datasets were collected but excluded entirely for analysis due to a sample size too small for statistical analysis (N=2 and N=1 stunted children after filtering). No other datasets which matched our inclusion criteria for age and stunting were analyzed.

Randomization	Randomization is not relevant to our study design because there was nothing to randomize.
Blinding	The experiments were not blinded. Blinding was not practical given that normally a single person was performing a bacterial growth experiment and necessarily had to know which group was which in order to perform the experiment.

Reporting for specific materials, systems and methods

We require information from authors about some types of materials, experimental systems and methods used in many studies. Here, indicate whether each material, system or method listed is relevant to your study. If you are not sure if a list item applies to your research, read the appropriate section before selecting a response.

Materials & experimental systems

n/a	Involved in the study	
☒	☐	Antibodies
☒	☐	Eukaryotic cell lines
☒	☐	Palaeontology and archaeology
☒	☐	Animals and other organisms
☐	☒	Human research participants
☒	☐	Clinical data
☒	☐	Dual use research of concern

Methods

n/a	Involved in the study	
☒	☐	ChIP-seq
☒	☐	Flow cytometry
☒	☐	MRI-based neuroimaging

Human research participants

Policy information about studies involving human research participants

Population characteristics	As described in the methods, microbiome data were analyzed from stunted and non-stunted children aged 2-5 years old. Most of this data was previously published. New sequencing data from populations in Antananarivo, Madagascar and Bangui, Central African Republic were obtained from the Afribiota project (Vonaesch et al 2018 a, b). Children at both study sites in the Afribiota population were matched by age and gender for each stunting status. The median age of the Madagascar population was 41.3 months, 53.0% were female and the median height-for-age z-score was -1.9. The median age of the CAR population was 40.3 months, 50.8% were female, and the median height-for-age z-score was -1.7.
Recruitment	The full recruitment procedure of the Afribiota study has been published: Vonaesch et al (2018). "Identifying the etiology and pathophysiology underlying stunting and environmental enteropathy: study protocol of the AFRIBIOTA project." BMC pediatrics 18(1) doi:10.1186/s12887-018-1189-5. Children with signs of acute infection (diarrhea, fever) or with recent antibiotics use were excluded from the study. Therefore, we may miss some pathogenic bacterial signatures associated with true infection. Children were also excluded from the Afribiota study if they were HIV positive or suffered from acute malnutrition (WHZ ≤-2). Therefore, our results may not be applicable to these populations.
Ethics oversight	The study protocol for Afribiota was approved by the Institutional Review Board of the Institut Pasteur (2016-06/IRB), the National Ethical Review Boards of Madagascar (55/MSANP/CE) and the Central African Republic (173/UB/FACSS/CSCVPER/16), and the Human Ethics Board of the University of British Columbia (H18-01108).

Note that full information on the approval of the study protocol must also be provided in the manuscript.
